# The contact hypothesis and the virtual revolution: Does face-to-face interaction remain central to improving intergroup relations?

**DOI:** 10.1371/journal.pone.0292831

**Published:** 2023-12-08

**Authors:** Julian Bond, John Dixon, Colin Tredoux, Eleni Andreouli

**Affiliations:** 1 School of Psychology and Counselling, Open University, Milton Keynes, United Kingdom; 2 Department of Psychology, University of Cape Town, Cape Town, South Africa; National Taiwan University, TAIWAN

## Abstract

Research on the contact hypothesis has traditionally prioritized the role of positive, direct, face-to-face interactions in shaping intergroup prejudices, but it has recently expanded to study indirect vicarious, negative, and online contact experiences. In the majority of studies though, there has been little direct comparison of the relationship between these different forms of contact and prejudice. The present research set out to compare the amount and effects of negative, online, and vicarious contact in the context of positive, face-to-face and direct contact in two studies. Study 1 comprised a national cross-sectional survey of relations between White and Black UK residents (n = 1014), and Study 2 comprised a national longitudinal survey of relations between Catholic and Protestant residents of Northern Ireland (n = 1030). The results of both studies indicated that positive face-to-face contact occurred more frequently and had a comparatively stronger relationship with prejudice than other forms of contact. However, they also indicated the effects of online, negative and vicarious forms of contact existed independently of those of direct, positive face-to-face contact. Moreover, online negative contact generally had a stronger relationship to prejudice than negative contact experienced face-to-face. Exploratory mediation analyses suggested the affective pathways from contact to prejudice may vary for different forms of contact.

## Introduction

The contact hypothesis states that tensions between two groups can be reduced if members of one group have positive contact with members of the other [1]. Pettigrew and Tropp’s influential meta-analysis of over 500 contact studies [2] provided strong evidence supporting this hypothesis. They reported that contact was associated with prejudice reduction in 94% of studies and that Allport’s so-called ‘optimal conditions’ (e.g., that groups involved in contact should be equal in status) strengthened this association [3]. They reported, too, that the effects of contact tended to generalise beyond local interactions to shape wider patterns of intergroup attitudes across a range of social settings. Although highlighting that the contact-prejudice relation is typically small in magnitude (on average around r = -0.21) and acknowledging the need to know more about the consequences of negative as well as positive contact experiences, Pettigrew and Tropp (re)established the contact hypothesis as one of social psychology’s best-evidenced interventions to promote social change [2]. Indeed, their review concluded that: “Given the current state of the research literature, there is little need to demonstrate further contact’s general ability to lessen prejudice” [2].

Like the vast body of work on intergroup contact, however, Pettigrew and Tropp’s review [2] privileged the role of direct, face-to-face interactions between members of different groups. More recently, researchers have sought to explore the role of other kinds of contact experiences, including extended contact (contact known to occur within an individual’s extended social network rather than contact experienced face-to-face [4]), vicarious contact (contact observed rather than experienced face-to-face [5]), imagined contact (contact that participants think about or anticipate rather than experience face-to-face [6]), and, most important to the present paper, online contact (contact that occurs in virtual, online spaces rather than being experienced face-to-face [7]).

Sometimes these alternative forms of contact have been treated as significant in their own right. For example, Mancini and Imperato [8] suggested that the “Internet can foster even more intimate relationships than face-to-face communication…making it an excellent tool to intergroup contact”. Similarly, Fiona White and colleagues [9] stated: “we argue that indirect contact is more than just a “simple” replacement for direct contact”. Often, however, alternative forms of contact have been framed as secondary, supplementary, or intermediary experiences, useful mainly in contexts where face-to-face interactions are difficult to implement (e.g., highly segregated societies) [10] but ultimately no substitute for interactions in which group members involved are physically co-present and directly interacting. Thus, for example, imagined, extended and online contact experiences have repeatedly been framed as ‘stepping stones’ on the path to establishing ‘actual’ interactions between members of historically divided communities. In sum, there is an implicit–and often explicit—assumption in the contact literature that the linkage between outgroup contact and outgroup attitudes and relations are especially significant for face-to-face interactions in comparison to indirect forms of contact (e.g., online). As an example, Brown and Paterson state [11]: ‘Most research has found that extended contact has weaker effects than direct contact, especially when both forms of contact were included in the same analysis’, indicating a clear privileging of face-to-face contact.

The present research explores the validity of this assumption. Firstly, it measures the amount of contact across different formats in order to understand the relevance of online contact in relation to face-to-face contact in people’s day-to-day lives. Secondly, it systematically compares the interrelations between different forms of online and face-to-face contact experiences and self-reported prejudice. Our starting position is that online contact is of rapidly increasing significance but that we still know relatively little about its comparative consequences for intergroup attitudes, stereotypes, and behaviours. In the same year that Pettigrew and Tropp’s seminal review was published [2], Facebook was launched to everyone over the age of 13 with a valid email address. Subsequently, Twitter, YouTube, WhatsApp, Snapchat, and many other virtual platforms have rapidly become integral to everyday communications between millions of users in almost every society on earth. This social media revolution has fundamentally altered how many of us interact with members of our own and other communities. Even before the beginning of the Covid pandemic in 2020, over three quarters of all Americans reported communicating regularly online [12]. Additionally, two out of three American teenagers now prefer to communicate online rather than face-to-face [13]. Among Gen Z and millennials internationally, 65% of people communicate digitally more frequently than in person, with the figure in the US and UK being even higher, at 74% [14].

In this rapidly changing communicative context, we would argue for a systematic and ongoing interrogation of the assumption that face-to-face interactions have primary significance in terms of their linkage with measures of intergroup relationships such as prejudice, and that virtual forms of interaction are either of lesser importance or merely conduits for encouraging ‘real’ contact. We would also argue for exploration of whether different forms of contact work via the same affective mediators. To that end, we present the findings of two studies designed to compare the relationship between varying forms of online and face-to-face intergroup contact (negative and positive; direct and vicarious) and intergroup prejudice. Study 1 comprises a national cross-sectional survey of relations between White and Black UK residents, and Study 2 comprises a national longitudinal survey of relations between Catholic and Protestant residents of Northern Ireland. The different contact forms are discussed in the next sections.

### Online contact

The increasing use of social media for communication raises questions that remain under-explored in the contact literature. Are positive and negative contact experiences more likely to occur in online or face-to-face contexts and with what consequences for intergroup attitudes and behaviours? Do online contact experiences exert greater or lesser impact on prejudice than face-to-face contact experiences? Alternatively, does the relationship between online communication and prejudice disappear if the effects of–arguably more fundamental—face-to-face contact are factored in? Are the theoretical mechanisms that underpin the effects of face-to-face and online contact isomorphic or do they implicate different predictors, mediators, moderators, and explanatory pathways?

The available evidence does not provide clear or complete answers to these questions, and the present research addresses this gap. In particular, relatively few psychological studies have explored the relationship between prejudice and the *naturally occurring* forms of online intergroup contact experienced by individuals in their everyday lives. Moreover, little research has systematically compared their impact with that of positive and negative face-to-face intergroup contact experiences. Most existing research has instead focussed on the impact of online intergroup contact experiences in isolation, and particularly on positive online contact unfolding in relatively controlled virtual environments [10–17]. This research has proved valuable in establishing the potential benefits of carefully engineering virtual spaces that encourage cooperative interaction between different groups.

In their chapter overviewing research on online contact, for instance, Hasler and Amichai-Hamburger [18] focused largely on artificially constructed contact experiences designed to encourage interactions in contexts where face-to-face exchanges are challenging (e.g., between Jews and Palestinians in Israel). Their review demonstrated that under the ‘right’ conditions computer mediated intergroup contact can have beneficial effects. Indeed, they showed that online environments are sometimes especially conducive to fostering the conditions that Gordon Allport hypothesised were optimal for contact to be effective [3]. In a study among American students, Dubrovsky et al. [19] found that text-based virtual contact increased status equality, perhaps because of its lack of visual cues. Online environments can also be structured in ways that readily encourage collaboration towards superordinate goals [20], and the ease and low cost of their creation can facilitate institutional support from relevant authorities [7].

Understandably, then, in line with classic contact theory, authors such as Hasler and Amichai-Hamburger [18] have been eager to demonstrate the positive potential of online contact as a vital first step in prejudice reduction. In their more recent meta-analysis, Imperato et al. [21] have confirmed that online contact has the potential to reduce prejudice. However, their review also highlights some limits in the available evidence. First, most studies consist of experiments conducted under controlled conditions, and there is limited evidence on the nature and consequences of naturally occurring online contact encounters of the kind that millions of people experience in everyday life—although there are some exceptions, including a survey of a national sample of Israeli Jews [22], and a survey of undergraduate students from Serbia, Cyprus, and Croatia [23].

Second, the affective valence of online contact, which is potentially vital in determining its relationship with prejudice reduction, has not been systematically explored. As such, existing research has arguably left unanswered some broader questions about the impact of online contact beyond the idealised and carefully monitored spaces of psychological experimentation [24].

### Contact valence

Negative contact has been significantly under-researched compared to positive contact. Although Allport was clearly aware of the dangers of negative interactions when formulating the contact hypothesis “…acquaintance may depreciate a person’s standing if it brings to light realistic defects in his nature” [3], until recently researchers have focused overwhelmingly on positive interactions. Over the past decade or so, however, research on the significance of contact valence has burgeoned. Based on their ongoing research program, for example, Fiona Barlow, Stefania Paolini and colleagues have advanced three main claims [25–27]. First, negative contact occurs far less frequently than positive contact in everyday life. Second, at least in some contexts, negative contact may nevertheless have a comparatively stronger effect; that is, it may increase prejudice more than positive contact decreases it. Third, this may be because negative interactions with members of other groups are more likely to produce attitudinal changes that generalise beyond the immediate context of interpersonal interaction to shape feelings about the outgroup as a whole.

More recent research has qualified some of these claims. For example. Using a meta-analytical approach, Paolini and McIntyre [28] suggested that the relative impact of negative versus positive contact varies depending on whether the outgroup is stigmatized or admired. In their 2021 review, Schäfer et al. [29] concluded that the comparative effects of negative and positive contact on prejudice remains unclear, with several recent studies reporting mixed or null findings, and some suggesting that positive contact sometimes has a larger effect on prejudice than negative contact. However, one empirical finding has emerged in almost all studies to date. There is now broad agreement that positive contact occurs more frequently than negative contact, even among minority groups (e.g., UK and German LGBTQ+ students [30], groups in conflict situations (e.g., Northern Irish students [31]) or groups that experience contact indirectly (e.g., Turkish Cypriot adults [27]. Schäfer et al. treat this core finding as a source of optimism because, among other reasons, frequent positive contact experiences may buffer against the effects of subsequent, less frequent, negative contact experiences.

As in other areas of contact research however, research on the negative-positive contact asymmetry has prioritized face-to-face interactions, and the degree to which existing findings apply within online environments remains uncertain. White et al. [32], for instance, argue that in online environments there is much greater potential for contact to become negative. Factors such as anonymity and lack of communicative accountability may have a disinhibiting effect, increasing the frequency and intensity of antagonistic interactions [33]. This idea resonates with the popular stereotype of ‘keyboard warriors’–people who transmit hateful content in a way that they would not do face-to-face.

There is importance evidence from research on racial microaggressions that occur online. In a study of American adolescents, Tynes et al. [34] (2008) found that most individuals of all ethnicities claimed to have experienced such microaggressions. Related work suggests that historically disadvantaged communities may be disproportionately targeted. Data from the Oxford Internet Survey [35] found that 39% of Black people in the UK reported experiencing cruel or hateful online contact, compared to 27% of White people. Moreover, there was a larger disparity in receipt of racially abusive emails, with 41% of Black people saying that they had received them compared to 8% of White people.

In summary, if intergroup contact is to have a beneficial effect on society overall, then it is important to explore the extent to which naturally occurring positive online contact—comprising millions upon millions of social exchanges every day—can drive prejudice reduction. Yet one of the underlying implications of experimental research on mediated online contact is that researchers must often take careful steps to ensure that the groups interact in a positive and respectful way [10]. As Hasler and Amichai-Hamburger acknowledge in their review, without active moderation of interactions online intergroup contact can easily descend into negative, hostile or even hateful interactions that potentially intensify rather than reduce prejudice. Such effects may be exacerbated by the fact that online exchanges are experienced not only directly by contact participants but also indirectly by potentially numerous onlookers within virtual spaces such as social media.

### Vicarious contact

When Gordon Allport proposed contact as a way of reducing intergroup prejudice [3], his focus was on direct contact between individuals from different groups. However, he also speculated on whether similar effects could be achieved via indirect exposure, e.g., to media representations of outgroup members “we know that advertising and films have molded our national culture to a considerable degree. May they not profitably be used in the task of remolding it?”.

Early work looked at parasocial contact [36] where people observed outgroup members at a distance, for example on TV. Later work focussed more on extended contact (e.g., [4]) gauging individuals’ knowledge of friendships between fellow ingroup members and members of an outgroup. More recently, the indirect observation of an interaction between ingroup and outgroup members has been described as *vicarious contact* [37] and a range of studies have confirmed that such contact has the ability to affect intergroup attitudes ([5,38–40]. The claim that vicarious positive contact has positive effects has been supported by Lemmer and Wagner [41], whose meta-analysis suggested that such contact is similar in effect to face-to-face contact, is long lasting, and may generalize to other outgroups.

Few studies have compared the effects of vicarious contact in online environments with other forms of contact, which is one objective of the present research program. An important exception is work by Brendesha Tynes and colleagues [34], who found that online *vicarious* negative contact was experienced significantly more frequently than online *direct* negative contact. This may be because individuals use social media and related online environments as onlookers as well as participants, thus any given instance of intergroup contact may be witnessed by multiple viewers beyond the immediate context of interaction. For example, instances of racial or sexual abuse on Twitter may reach not only large numbers of onlooking followers, but also, via retweeting, a much wider audience. Vicarious online contact, in short, has the potential to powerfully influence intergroup attitudes and stereotypes even if it has not been a central focus within the literature on the contact hypothesis.

### Contact between historically advantaged and disadvantaged groups

The majority of studies on contact theory have been conducted among members of the majority / advantaged group. This has partly been for practical reasons (e.g., achieving sample size), and partly because of the assumption, underlying most work on prejudice, that intergroup relations can be best improved by reducing prejudice and discrimination of the advantaged.

In a meta-analytic study of contact effects, Pettigrew and Tropp found that the effectiveness of contact was significantly greater among majority/advantaged samples than among the minority/disadvantaged samples [2]. Because the present research was designed to understand whether there are different levels of effect between online and face-to-face contact it also seemed logical to test whether any differences varied between majority/advantaged and minority/disadvantaged samples.

### Overview of the present research

Given rapid changes in the nature and media of everyday human communication, our studies explore whether the historic tendency to privilege the effects on prejudice of face-to-face direct contact over other forms of contact remains justified. To do so, it systematically contrasts the effects of several modes of intergroup contact (online and face-to-face, negative and positive, direct and vicarious) across two forms of intergroup relations, namely race relations in the UK (Study 1) and sectarian relations in Northern Ireland (Study 2). In doing so, our research questions are both descriptive (e.g., what is the self-reported frequency of varying modes of contact across these two forms of intergroup relations?) and comparative (e.g., what is the relative strength of the relationship between different modes of online and face-to-face contact and indicators of prejudice). In Study 2, we also explore whether varying forms of contact are explicable via the same theoretical mechanism or whether there are different mediators pertaining to different contact forms.

### Study 1

While contact research has explored a variety of kinds of intergroup relations, most work has focussed on the effectiveness of face-to-face contact in reducing ethnic and racial prejudices. In the UK, where study 1 was conducted, interracial contact currently also has heightened resonance recently due to the global prominence of the Black Lives Matters movement [42], and associated protests in many local cities [43]. By sampling both Black and White residents of the UK, Study 1 was also able to explore the potentially variable relationship between different forms of face-to-face and online and prejudice across racial groups. Research conducted in the US suggests, for example, that positive intergroup contact is more effective at reducing prejudice among the White majority rather than the Black minority [44].

## Method

### Participants and procedure

The survey was cross-sectional and conducted using an online questionnaire, with the sample being recruited from a national access panel across Great Britain. Written consent for the study was given by the Open University’s Human Research Ethics Committee. All respondents had internet access, ensuring they had the opportunity to experience online as well as face-to-face interaction. A total sample of 1030 adults were recruited who self-reported as 501 Black and 529 White. Respondents were informed of the nature of the study and all respondents had to confirm that they had read the study information and that they were happy to take part. This consent was achieved online. The Black sample average age was 37.7 (SD = 14.1), with 57% female and 43% male respondents. The White sample average age was 45.2 (SD = 16.8), with 52% female and 48% male respondents.

### Measures

**Contact.** Because the study itself was an online survey, and measured the frequency of different forms of self-reported online and face-to-face contact (positive and negative; direct and vicarious), it was important to keep the measures of contact as simple as possible to avoid participant fatigue. This was achieved by using a variation of a single item contact scale employed successfully in previous research [26]. This consisted of a seven-point Likert scale (e.g., “On average, how often do you have negative / bad face-to-face direct contact with Black/White people?”), which was adapted across different forms of contact (see online supplementary material for a full list of items measuring this and all other variables in the two studies).

**Prejudice.** Prejudice was measured using the approach of Paolini et al. [31]. This consisted of an overall feeling thermometer, which asked respondents to put an X on an eleven-point scale ranging from extremely unfavourable to extremely favourable, and a set of six five-point semantic differential scales (e.g., warm → cold). This had a McDonald’s ω of .85 for the Black sample and .91 for the White sample.

## Results

### Frequency of self-reported contact across different contact forms

**Fig 1:**
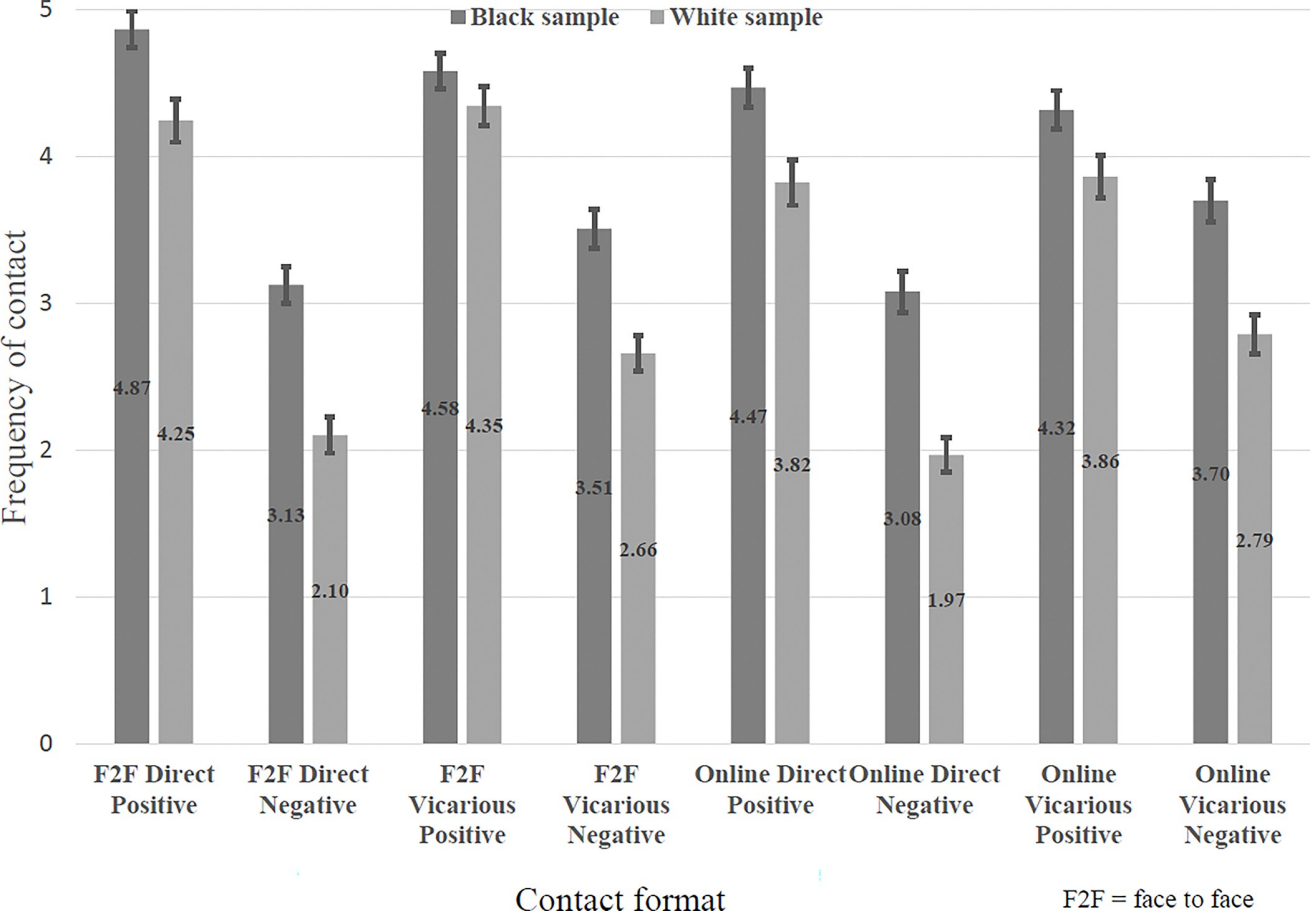
Mean frequency of different forms of online and face-to-face contact.

The first set of analyses focus on the amount of contact reported across different contact formats. Tables showing the values, standard deviations and intercorrelations between the different contact forms can be found in the supplementary material. Fig 1 shows the levels of contact by the eight contact types, with the error bars indicating 95% confidence intervals. The data are expressed as mean contact frequency scores on a scale ranging from 1 (‘never’) to 7 (‘extremely frequently’). Using a two-way Analysis of Variance in GLM, SPSS v. 27 with Type III sums of squares, the results show that the Black sample has significantly more intergroup contact than the White sample (F(1,1029) = 26.192, p < .005, average partial η^2^ = 0.06). In addition, this difference is significantly greater for negative contact compared to positive contact (t(1028) = 4.70, p < .001, Cohen’s d = 3.80). This provides additional statistical support for analyzing the two samples separately.

In order to achieve a greater understanding of the interrelationships between the different forms of contact, we can think of the contact measures as varying across three factors, each with two levels: Face-to-face/Online, Positive/Negative, and Direct/Vicarious. On this basis, data were analysed with a three-way repeated measures Analysis of Variance in GLM, SPSS v. 27 with Type III sums of squares. The results are shown in Table 1.

**Table 1:** ANOVA table for model of contact frequency as a function of positiveness of contact (positive vs negative), immediacy of contact (face-to-face vs online), and directness of contact (direct vs vicarious).

	Black Sample				White Sample			
	Type III SS	df	F	p	Type III SS	df	F	p
Positive/Negative	1458.8	1,500	278.9	< .001	3023.376	1,528	544.8	< .001
Face-to-face/Online	16.6	1,500	14.4	< .001	54.669	1,528	42.0	< .001
Direct/Vicarious	20.1	1,500	18.3	< .001	152.365	1,528	121.6	< .001
Posneg * F2FOnl	40.7	1,500	35.5	< .001	53.764	1,528	51.4	< .001
Posneg * DirVic	130.1	1,500	105.6	< .001	101.996	1,528	95.9	< .001
F2FOnl * DirVic	8.6	1,500	10.2	0.001	2.807	1,528	3.9	0.049
Posneg * F2FOnl * DirVic	0.7	1,500	0.9	0.334	6.909	1,528	9.1	0.003

df = degrees of freedom.

Despite the differences in the absolute amount of contact, and the average valence of contact between the two populations, the same patterns are seen for the main effects and two-way interaction for both samples. In each case the three main effects and the three two-way interactions are all statistically significant. For the main effects there is more positive than negative contact, more face-to-face than online contact and more vicarious than direct contact.

The strongest two-way interaction effect sizes (η_p_^2^) are those linked to the interaction between valence and contact format; all of them are either medium or large effects [45]. Although statistically significant the effect sizes for the interaction between online/face-to-face and direct/vicarious are either small or zero and so are not analyzed in more detail.

**Fig 2:**
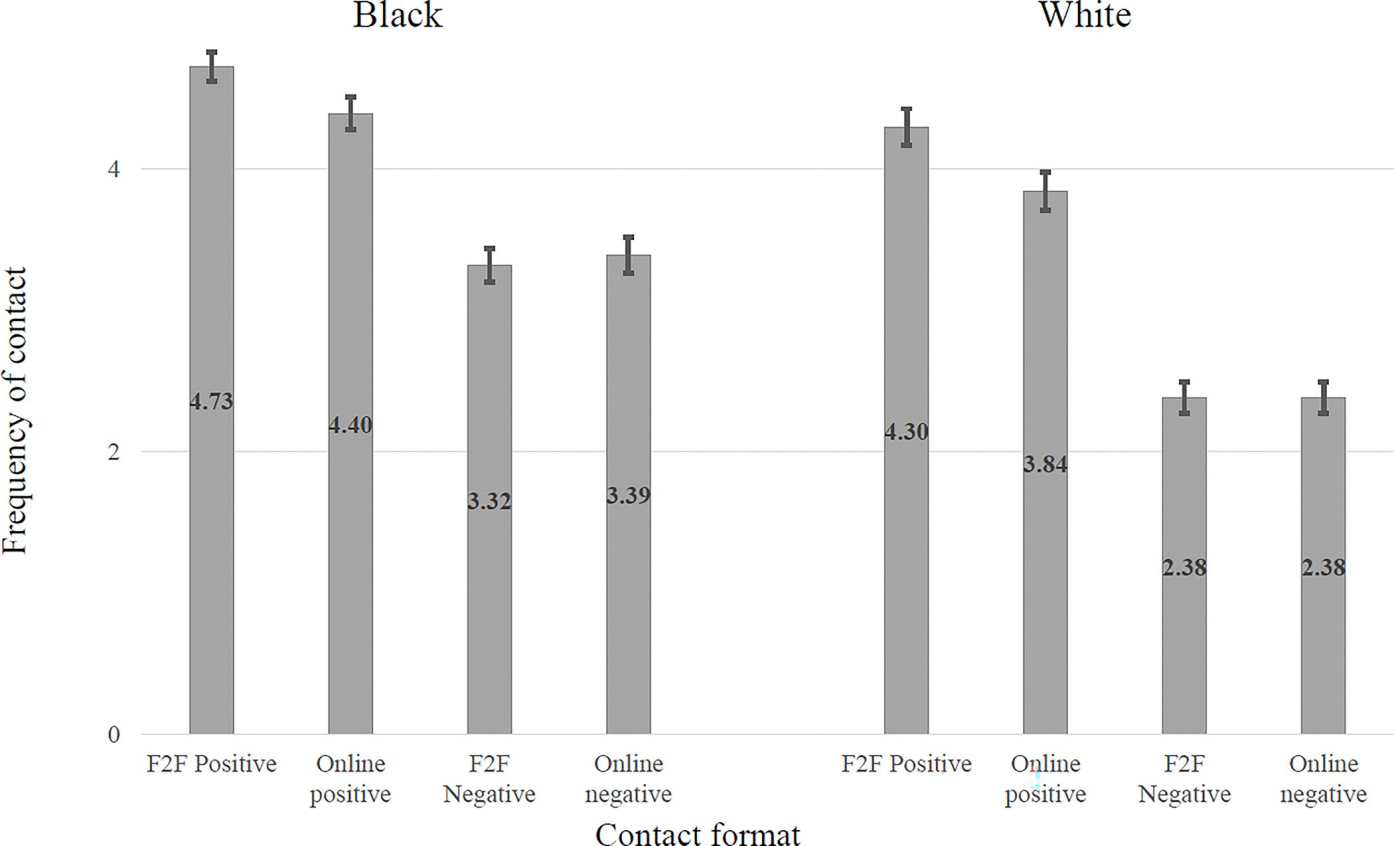
Differences in self-reported frequency of contact according to valence (positive vs negative) and immediacy (face-to-face vs. online) of contact in national Black (N = 501) and White (N = 529) samples of UK adults.

The relationship between valence and whether the contact was face-to-face or online is shown in Fig 2. This shows that for both samples everyday contact is more positive when it is face-to-face than when it is online. In each sample there is significantly more *positive* contact face-to-face than online (t(500) = 6.79, p < .001, Cohen’s d = .18 for the Black sample, t(529) = 8.54, p < .001, Cohen’s d = .36 for the White sample). Conversely there is no significant difference between *negative* contact face-to-face and *negative* contact online (t(500) = -.1.55, p = .122, Cohen’s d = .05 for the Black sample and t(529) = 0.05, p = .963, Cohen’s d = 0 for the White sample).

**Fig 3:**
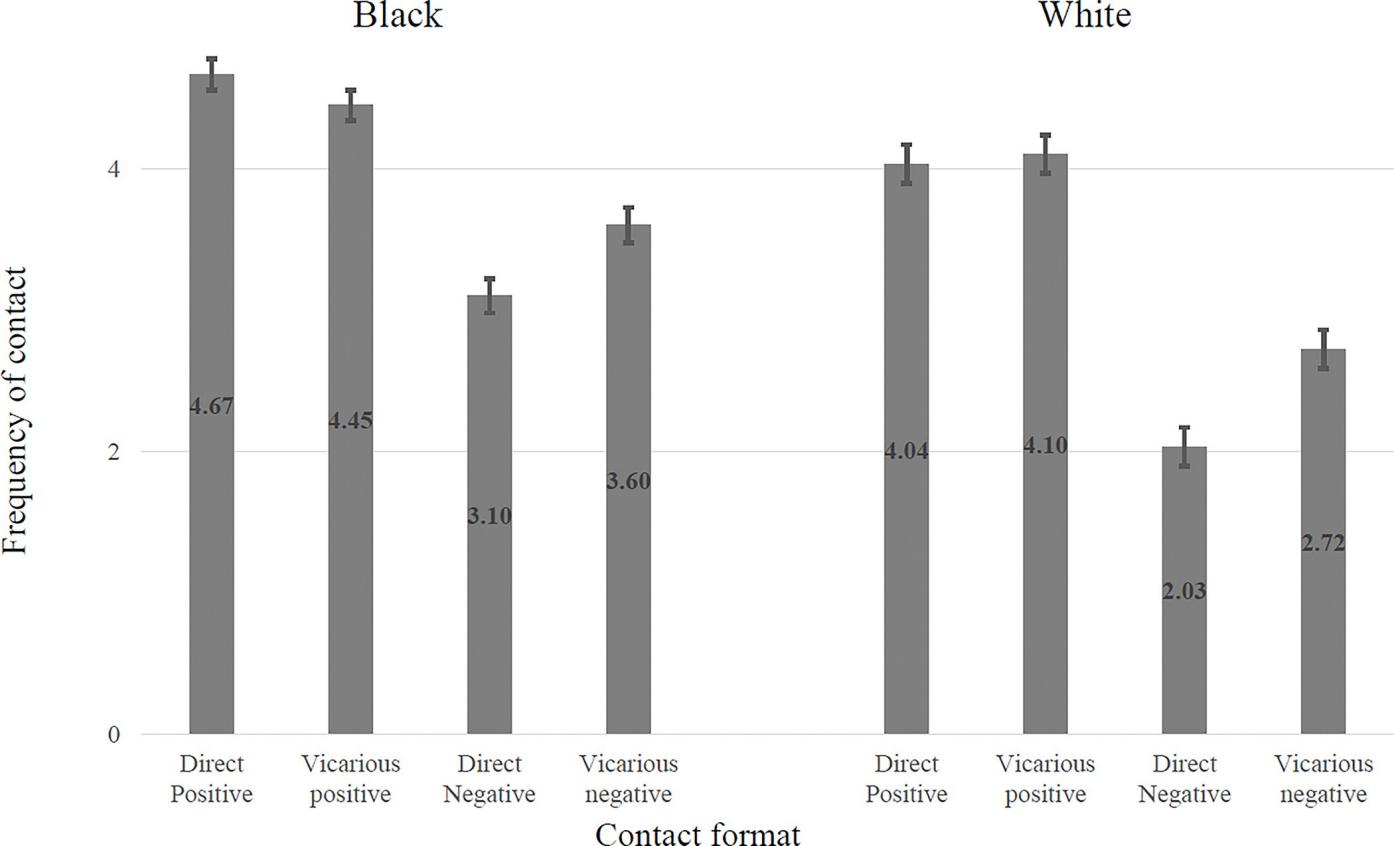
Differences in self-reported frequency of contact according to valence (positive vs negative) and directness (direct vs. vicarious) of contact in national Black (N = 501) and White (N = 529) samples of UK adults.

The equivalent relationship between valence and whether the contact was direct or vicarious is shown in Fig 3. This shows that for both samples everyday contact is more positive when it is direct than when it is vicarious. In each sample there is significantly more *negative* contact when the contact is vicarious than when it is direct (t = -10.49, p < .001, Cohen’s d = .36 for the Black sample, t = -14.69, p < .001, Cohen’s d = .52 for the White sample). Conversely, there is significantly more *positive* contact when the contact is direct than when it is vicarious in the Black sample (t = 4.50, p < .001, Cohen’s d = .17) and no significant difference between valences for the White sample (t = -1.48, p = .140, Cohen’s d = .04).

### Relationship between self-reported contact and prejudice

Having established that the self-reported frequency of contact varies across different forms of contact, the next stage of the analysis examines the relationships between these forms of contact and prejudice. Details of the latent variable construction are provided in the online supplementary material (see OSM4). The analysis was conducted in SPSS v.27. As a starting point, Table 2 shows the bivariate correlations between contact types and prejudice. Following the approach of Paolini et al. [31], the prejudice variable was created from the six Semantic differential scales and the feelings thermometer rescaled to match the scoring of the other scales (i.e. 1 was scored as 1 and 11 was scored as 5 with equidistant gaps between the levels).

**Table 2:** Bivariate correlations between valence, immediacy, and directness of contact, and prejudice, in a national sample of Black (N = 501), and White (N = 529) adults.

	Black sample				White sample			
			95% confidence interval				95% confidence interval	
Contact format	r	p	Lower	Upper	r	p	Lower	Upper
Positive face-to-face direct	-0.368	< .001	-0.441	-0.290	-0.319	< .001	-0.393	-0.240
Negative face-to-face direct	0.225	< .001	0.140	0.306	0.305	< .001	0.225	0.380
Positive face-to-face vicarious	-0.255	< .001	-0.335	-0.171	-0.308	< .001	-0.383	-0.228
Negative face-to-face vicarious	0.185	< .001	0.099	0.268	0.234	< .001	0.152	0.313
Positive online direct	-0.269	< .001	-0.348	-0.186	-0.262	< .001	-0.340	-0.181
Negative online direct	0.247	< .001	0.162	0.327	0.261	< .001	0.180	0.339
Positive online vicarious	-0.237	< .001	-0.318	-0.153	-0.262	< .001	-0.339	-0.180
Negative online vicarious	0.228	< .001	0.143	0.309	0.071	0.101	-0.014	0.156

All correlations were significant (p< .001) except the relationship between prejudice and negative online vicarious contact among the White sample. With the exception of negative online vicarious contact in the White sample, the correlations are all small to medium in size. All significant correlations are in the expected direction (i.e., all forms of positive contact are negatively correlated with prejudice and all forms of negative contact are positively correlated with prejudice).

In both samples, the highest bivariate correlation occurs for positive face-to-face contact, although only in the Black sample is the difference statistically significant to the online equivalent (t = 2.48, p = .014 for the black sample, t = 1.613, p = .106 for the White sample). None of the other comparisons between the face-to-face and online equivalents are statistically significant apart from negative vicarious contact in the White sample where the face-to-face form has a significantly higher correlation (t = 4.127, p < .001). This latter result is discussed in more detail later in this section.

The next stage of analysis was designed to understand whether the less historically studied contact experiences (i.e., self-reported online and vicarious contact) are ‘stepping stones’ to ‘proper contact’ (i.e. face-to-face, direct) by testing whether they still have a significant relationship with prejudice even after the effect of face-to-face or direct contact has been removed.

To achieve this, in each case a two-stage hierarchical regression was conducted with the first independent variable being the form of contact typically measured in contact studies (face-to-face and direct) and the second independent variable being the less commonly measured form of contact (online and vicarious respectively). Table 3 shows initial R^2^ values and the additional R^2^ that is achieved by adding in the second predictor. When all 8 contact types are included then the regression yields R^2^ = .494 (F(8,492) = 19.85, p< .001), for the Black sample and R^2^ = .522 (F(8,520) = 24.33, p< .001) for the White sample.

**Table 3:** Hierarchical regressions of immediacy and directness of contact on prejudice, testing whether adding a contact format less frequently studied (online contact, vicarious contact) improves on a baseline model.

	Sample								
	Black sample					White sample			
	R^2^	Δ R^2^	F(Δ)	p(Δ)		R^2^	Δ R^2^	F(Δ)	p(Δ)
**F2F / Online**									
1. All F2F contact	0.20	0.20	30.96	< .001		0.23	0.23	39.98	< .001
2. All online contact	0.24	0.04	7.19	< .001		0.27	0.04	6.88	< .001
**Direct / Vicarious**									
1. All Direct contact	0.21	0.21	32.50	< .001		0.24	0.24	41.33	< .001
2. All Vicarious contact	0.24	0.04	5.91	< .001		0.27	0.03	5.81	< .001

Δ R^2^ is the uniqe contribution of each stage of the regression.

F(Δ) and p(Δ) test the statistical significance of the unique contribution.

Across both samples, the initial R^2^ values for face-to-face contact (0.20 and 0.23) are statistically significant (F(4,496) = 30.96, p < .001 for the Black sample and F(4,524) = 39.98, p < .001 for the White sample) as are the additions to the R^2^ values (0.04 and 0.04) achieved by adding online contact (F(4,492) = 7.19, p < .001 for the Black sample and F(4,520) = 6.88, p < .001 for the White sample). Similarly, across both Black and White samples, the initial R^2^ values for direct contact (0.21 and 0.24) are statistically significant (F(4,496) = 32.50, p < .001 for the Black sample and F(4,524) = 41.33, p < .001 for the White sample) as are the additions to the R^2^ values (0.04 and 0.03) achieved by adding vicarious contact (F(4,492) = 5.90, p < .001 for the Black sample and F(4,520) = 5.81, p < .001 for the White sample). In both cases the additional R^2^ from the second format was around 0.04. This represents a small to medium effect [46] and is similarly sized to the overall effect of contact (average r value of -.21) presented by Pettigrew and Tropp [2]. This demonstrates that the non face-to-face forms of contact such as online or vicarious contact are not only related to prejudice as would be expected from previous research but add additional predictive power to models seeking to explain the relationship between contact and prejudice over and above that achieved by models that are based on *historically privileged* face-to-face direct formats.

Having established that the alternative contact formats add significant additional predictive power to models explaining the relationship between contact and prejudice, prejudice was then regressed on the eight forms of contact with all the independent variables entered together (Table 4). This allows us to examine the eight different kinds of contact-prejudice relations at a more granular level.

**Table 4:** Multiple regression analysis of all eight contact formats on prejudice.

	SAMPLE										
	Black sample						White sample				
	β	SE(β)	t	p	VIF		β	SE(β)	t	p	VIF
Positive face-to-face direct	-0.27	0.05	-5.29	0.000	1.673		-0.17	0.06	-2.99	0.003	2.380
Negative face-to-face direct	0.08	0.06	1.46	0.144	2.079		0.21	0.05	3.85	< .001	2.111
Positive face-to-face vicarious	-0.08	0.05	-1.61	0.107	1.699		-0.07	0.06	-1.29	0.196	2.388
Negative face-to-face vicarious	0.00	0.05	-0.05	0.959	1.909		0.18	0.05	3.46	0.001	1.914
Positive online direct	-0.03	0.05	-0.56	0.578	1.851		-0.11	0.06	-1.96	0.050	2.301
Negative online direct	0.12	0.06	2.04	0.042	2.063		0.15	0.06	2.74	0.006	2.243
Positive online vicarious	-0.13	0.05	-2.49	0.013	1.738		-0.11	0.05	-2.07	0.039	2.019
Negative online vicarious	0.17	0.05	3.24	0.001	1.845		-0.13	0.05	-2.64	0.008	1.773

β = standardized coefficient.

Variance inflation measures (VIFs) were calculated to check on intercorrelation. Across all sixteen tests, the highest VIF value was 2.243. All values therefore are comfortably below recommended upper values and so there is no serious issue with multicollinearity [47]. Overall the regression models are highly predictive, achieving R^2^ = .494 for the Black sample (F(8,492) = 19.85, p< .001) and R^2^ = .522 for the White sample (F(8,520) = 24.33, p< .001).

For the Black sample, the greatest beta value is for positive face-to-face contact, but all the other significant betas are for online contact, with one of the positive and both negative formats being significant, suggesting an interaction between online / offline contact and valence. For the White sample, seven out the eight formats have a significant relationship with prejudice, with all the online beta values being significant and three out of the four face-to-face beta values being significant. This suggests that the impact of online and face-to-face contact varies between Black and White groups.

An unexpected result, however, is that we obtained a significant negative coefficient value for negative online vicarious contact for the White group, whereas we expected to find a positive coefficient after controlling for all other forms of contact we studied. In other words, negative online vicarious contact is associated with a *decrease* rather than *increase* in prejudice. This relationship contrasts with all other forms of negative contact across the two groups where, as would be expected, negative contact experiences are linked to an *increase* in prejudice.

## Discussion

Our results in Study 1 suggest that positive face-to-face, direct contact remains central to the reduction of racial prejudice. This form of contact was experienced more frequently than other forms of contact by both our Black and White respondents. Moreover, as Table 2 indicates, it had the strongest overall relationship with lower prejudice, a trend that broadly confirms the conclusions drawn in recent reviews of the field about the efficacy of the traditional contact hypothesis [2,48].

However, our results also reveal some interesting complexities and qualifications. First, although positive face-to-face direct contact had the strongest relationship with prejudice in our survey, other forms of contact also had independent, significant, and additive effects. Indeed, for both the Black and White samples, there was clear evidence that models that included online, negative and vicarious contact experiences as predictors explained significantly more variance in prejudice than models based solely on face-to-face, direct and positive forms of contact. In other words, even when controlling for the effects of positive face-to-face interactions, other kinds of interactions remained important in understanding the overall relationship of contact and prejudice.

Second, our data indicate that the relative proportion of negative and positive contact individuals’ experience may vary markedly across online and face-to-face contexts. Overall, as we have noted, our Black and White respondents both reported experiencing positive contact most frequently in the form of direct, face-to-face interactions. Conversely, however, they reported experiencing online negative contact just as frequently as face-to-face negative contact. Moreover, negative contact experiences in both online and face-to-face environments were more frequent for Black than for White respondents. As such, the global claim that negative contact occurs less frequently than positive contact [25,29] may need to be refined. We may need to recognise that the prevalence of negative interactions may itself display contextual variability across different kinds of online and offline environments and across different groups.

Their relationship with prejudice may also vary. For our Black respondents, for example, online but not face-to-face negative contact experiences were significantly associated with increases in prejudice within our multiple regression model (see Table 3). Online negative contact, in other words, may have a greater impact on Black racial attitudes and perceptions than face-to-face negative contact, an idea which resonates with research on the damaging consequences of online racial micro-aggressions [34], particularly for minority group members.

To conclude this discussion, we want to highlight an intriguing and arguably counter-intuitive finding, which concerns the relationship between online vicarious contact and prejudice in our White sample (see Table 4). In this case, observing negative interracial contact online was associated with a *reduction* rather than an *increase* in self-reported prejudice amongst the White population, a finding that runs contrary to some previous work [11,49]. We hypothesize that this finding may reflect the nature of the contact being observed. Watching vicarious interactions online in which Black people are mistreated, for example, may cue feelings of sympathy or support amongst the White population.

This is consistent with Vezzali et al.’s findings [50], which suggested that negative vicarious intergroup contact increased the likelihood of advantaged group members taking collective action in support of the disadvantaged group. It is also consistent with the findings of Lissitsa and Kushnirovich [51] who found that, among a sample of Israeli Jews, negative parasocial online contact with Arabs, was linked to a reduction in reported subtle prejudice rather than the expected increase.

## Study 2

Study 2 extended Study 1 in three ways. First, it sought to replicate study 1 in a different intergroup and societal context, namely sectarian relations in Northern Ireland. Since implementation of the “Good Friday Agreement” in 1998, which established a devolved government in Northern Ireland and officially ended over 30 years of armed conflict, relations between local Protestant and Catholic communities have generally improved and intergroup violence has decreased. However, the country remains highly segregated along sectarian lines, and political, religious and social tensions between the two communities persist. Various authors have argued that intergroup contact, including vicarious contact [52] and online contact [10], may be particularly important in improving relationships within this historically segregated society.

Second, study 2 employed a longitudinal design. It therefore addressed some of the methodological limits of the cross-sectional design used in Study 1 where, for example, the causal direction of contact-prejudice relations would be difficult to specify.

Third, in addition to the variables measured and analysed in Study 1, Study 2 also explored some variables that potentially mediate the contact-prejudice relationship. It has already been stated that there is relatively little research looking at online contact. What research exists is almost entirely focussed on demonstrating that online contact links to prejudice and does not seek to test whether the same mediators are involved. In study 2 we aimed to begin to explore whether different forms of online and face-to-face contact operate through different theoretical pathways.

With this objective in mind, a criterion for selecting the mediators was to choose variables where there was already clear evidence that they mediated the relationship between face-to-face contact and prejudice. Specifically, building on research conducted in Northern Ireland and more widely, we explored the role of three well-established affective mediators of the contact-prejudice relationship, namely symbolic threat, realistic threat, and anxiety about contact [5,17,48,53,54]. Our aim was to explore whether these mediators operate in the same way across different combinations of positive and negative and online and face-to-face forms of contact.

## Method

### Participants and procedure

As for Study 1, written consent for the study was given by the Open University’s Human Research Ethics Committee. The first stage of the survey was conducted using an online questionnaire, with the sample being recruited from a national access panel across Northern Ireland. As with Study 1, all respondents had internet access, enabling comparison between their online and offline contact. Prior to starting the survey, respondents were informed of the nature of the study and all respondents confirmed online that they had read the information and were happy to take part in the study. The total sample was 1014, (447 Catholics and 567 Protestants). The Catholic sample’s average age was 38.7 years (SD = 12.9) and comprised 65% female and 35% male respondents. The Protestant sample’s average age was 43.2 years (SD = 14.6) and comprised 61% female and 39% male respondents.

The second stage of fieldwork took place between 27^th^ March and 12^th^ April 2020, approximately 100 days after the first stage. In this intervening period, the Covid-19 pandemic had taken hold in the UK, and Northern Ireland, like many other societies, had been locked down to limit the spread of illness and death. Perhaps due to the challenges of lockdown, the size of the stage two sample, after data cleaning and cross period matching, was reduced to 249 participants. Comparing the sample for those who completed both stages and those who only completed stage 1, there are no structural differences. Tests were carried out to compare the key results (contact amounts and latent variable scores). For the Catholic sample there were no significant differences between the two groups, and for the Protestant sample there was one significant difference. With 22 tests being conducted this does not suggest a major difference between the two groups. Full details are included in the supplementary material (see OSM2).

It is also worth noting that despite lockdown, none of the mean scores for all 8 forms of contact at stage 2 was significantly different to the equivalent mean scores at stage 1 in either sample.

### Measures

The questions on contact and prejudice were the same as those used in Study 1 but adapted to focus on interactions between Protestants and Catholics in Northern Ireland.

### Mediators

Intergroup threat was measured using an adaptation of Stephan et al.’s scale [55]. The measure consisted of five items for each type of threat, measuring unfair treatment and competition for political and economic power for *realistic threat* and perceived differences in values and worldviews for *symbolic threat* on a scale from disagree strongly to agree strongly (i.e., a five point scale, see online supplementary material OSM1). These had McDonald’s ω values of 0.88 and 0.85 respectively for Catholics and .91 and .84 for Protestants. Intergroup anxiety was measured using a variation of Stephan and Stephan’s scale [56] adapted for the Northern Irish situation by Paolini et al. [31] (see online supplementary material OSM1). This used six of the original eleven items (happy, awkward, self-conscious, confident, relaxed and defensive) and measured anxiety about experiencing intergroup contact on a scale ranging from ‘Not at all’ to ‘Extremely’ This had McDonald’s ω values of .85 for Catholics and .87 for Protestants.

## Results

**Fig 4:**
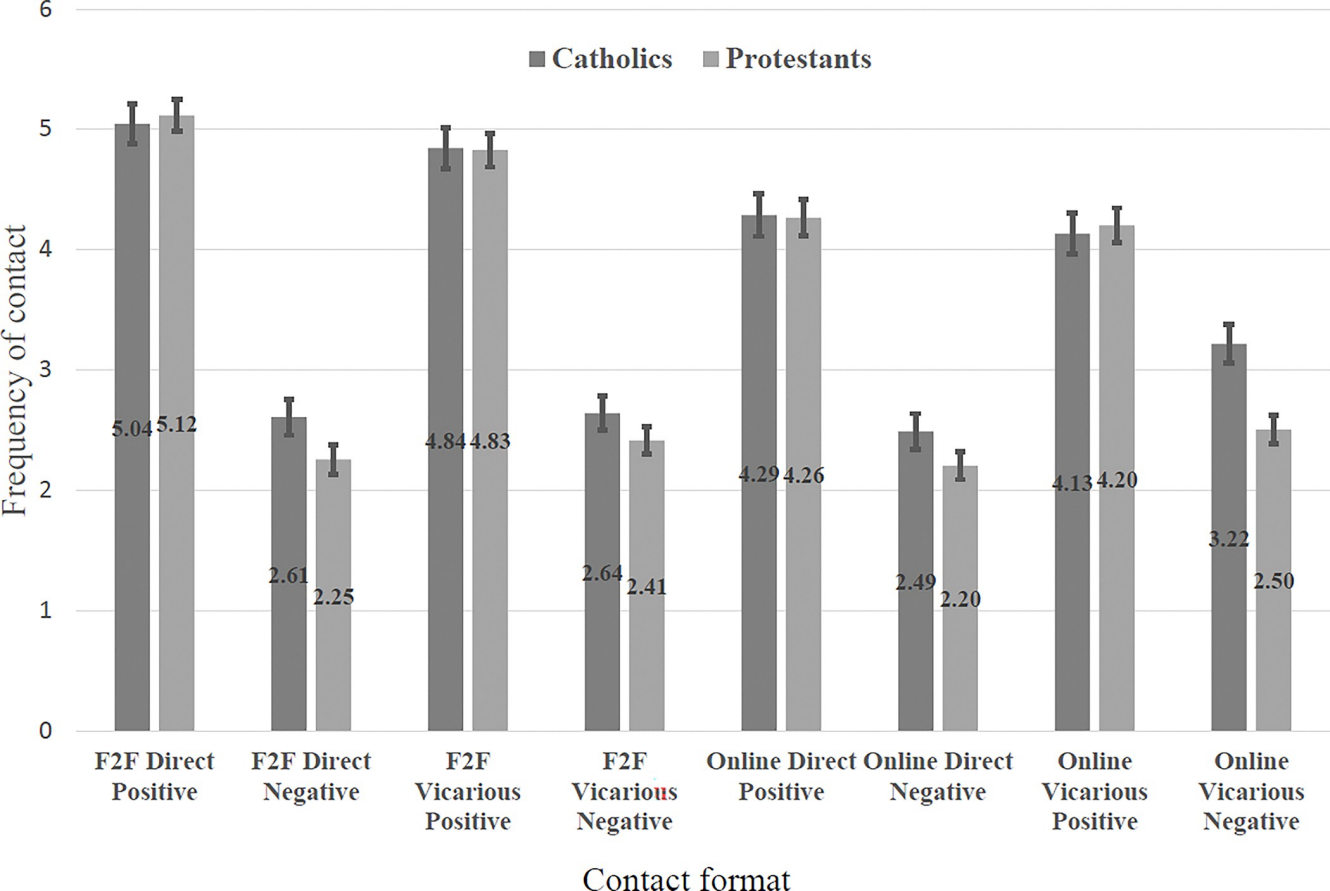
Mean frequency of different forms of online and face-to-face contact.

For the first section of results, the data are taken from stage one of the study, making them directly comparable with study one. We begin by considering the self-reported frequency of different forms of contact. Tables showing the values, standard deviations and intercorrelations between the different contact forms can be found in the supplementary material (see OSM3). Fig 4 displays mean frequencies of the eight contact types, with the error bars indicating the 95% confidence intervals. As in Study 1, the data are expressed as mean scores on a scale ranging from 1 ‘never’ to 7 ‘extremely frequently’.

Inspection of the descriptive statistics suggests that for all forms of self-reported contact (online versus face-to-face, direct versus vicarious), the amount of positive contact is significantly greater than the amount of negative contact. Moreover, face-to-face positive contact experiences are more prevalent than online positive contact experiences. It is again worth noting more subtle variations within respondents’ experiences of different forms of negative contact in Fig 4. For example, for both Catholic and Protestant respondents, online vicarious negative contact experiences seem to be more prevalent than any other form of negative contact experiences.

The next step in our analysis examined these differences in self-reported contact frequency between the different forms of contact more systematically, again considering three factors, each with two levels: Positive/Negative, Face-to-face/Online and Direct/Vicarious and using a three-way Analysis of Variance in GLM, SPSS v. 27 with Type III sum of squares. The results are shown in Table 5 and echo the findings of Study 1. All of the main effects and two-way interactions are significant except direct versus vicarious contact among Protestants.

**Table 5:** ANOVA analysis of contact frequency by format.

	Catholic					Protestant				
	Type III SS	df	F	p	η_p_^2^	Type III SS	df	F	p	η_p_^2^
Positive/Negative	3025.0	1,446	343.9	< .001	0.435	5781.4	1,566	773.5	< .001	0.577
Face-to-face/Online	56.9	1,446	36.5	< .001	0.076	146.4	1,566	89.2	< .001	0.136
Direct/Vicarious	9.4	1,446	8.2	0.004	0.018	0.8	1,566	0.9	0.347	0.002
Posneg * F2FOnl	207.3	1,446	122.9	< .001	0.216	163.4	1,566	115.2	< .001	0.169
Posneg * DirVic	69.6	1,446	65.8	< .001	0.129	46.9	1,566	46.2	< .001	0.076
F2FOnl * DirVic	31.0	1,446	32.0	< .001	0.067	9.4	1,566	11.8	0.001	0.020
Posneg * F2FOnl * DirVic	23.4	1,446	20.7	< .001	0.044	0.5	1,566	0.7	0.391	0.001

df = degrees of freedom.

For the main effects there is significantly more positive than negative contact in both samples, significantly more face-to-face than online contact in both samples and more vicarious than direct contact, although this difference is only significant in the Catholic sample.

The effect size of the valence effect is very high in both samples, the effect size of face-to-face versus online is medium for Catholics and high for Protestants while the effect size for direct versus vicarious contact is low in both samples.

As with Study 1, the two-way interactions involving contact valence have the greatest effect size with high η_p_^2^ figures for the valence and face-to-face / online interaction in both samples and medium η_p_^2^ figures for the valence and direct / vicarious interaction in both samples. Although the effect sizes for the interaction between face-to-face / online contact and direct/vicarious contact are slightly greater than for study 1, they are still significantly lower than the other two-way interactions and so are not analyzed in greater detail.

**Fig 5:**
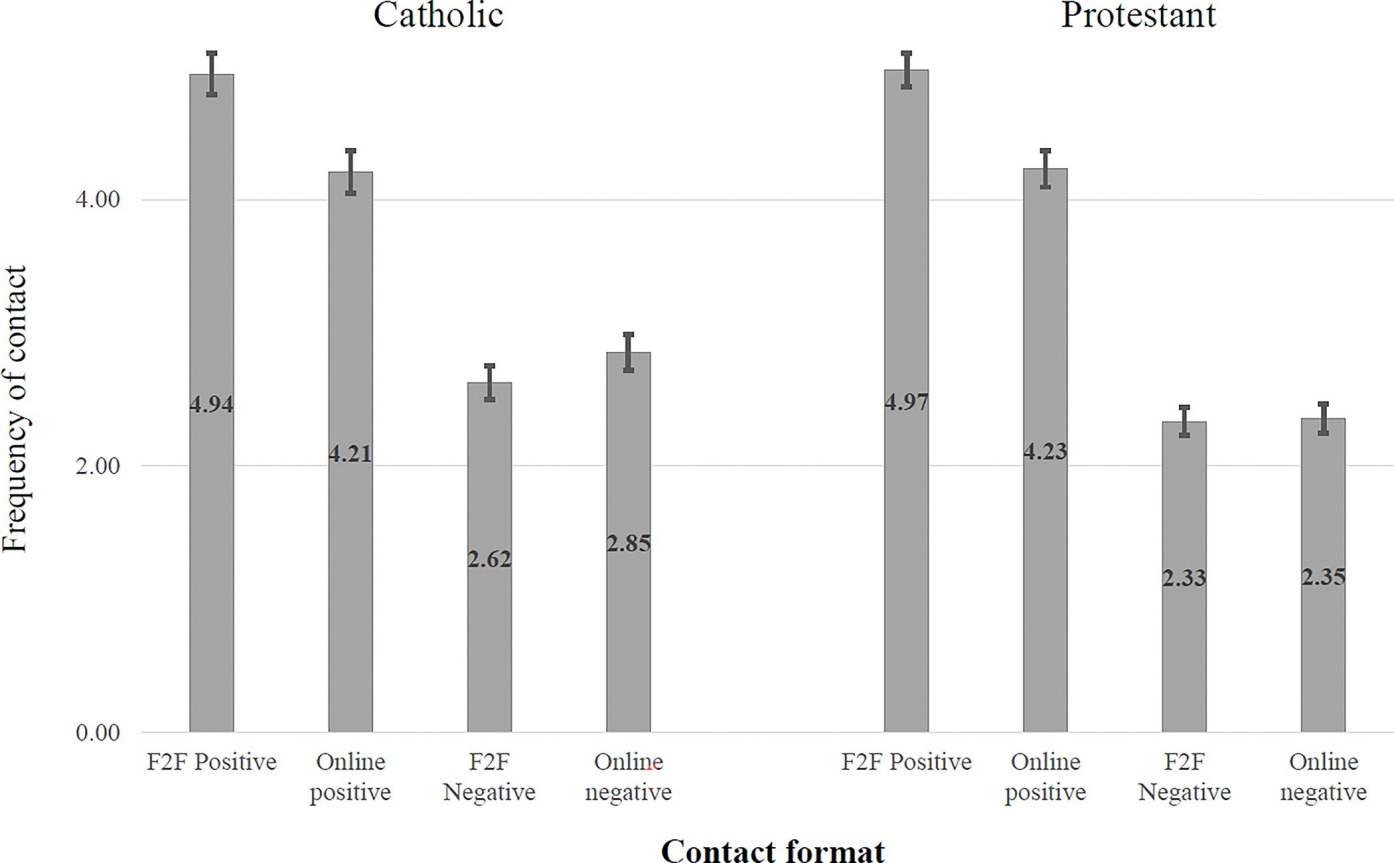
Two-way interaction effects between valence and face-to-face/online contact.

Fig 5 shows the results of the analysis of the two-way interaction between valence and whether the contact was online or offline.

In both samples there is significantly more *positive* contact face-to-face than online (Catholic sample, t(446) = 12.05, p < .001, Cohen’s d = .43, Protestant sample (t = 12.37, p < .001, Cohen’s d = .46)). Conversely in the Catholic sample there is significantly more *negative* contact online compared to face-to-face (t = .3.85, p < .001, Cohen’s d = .16), while for the Protestant sample there is no significant difference (t = -0.48, p = .635, Cohen’s d = .02).

**Fig 6:**
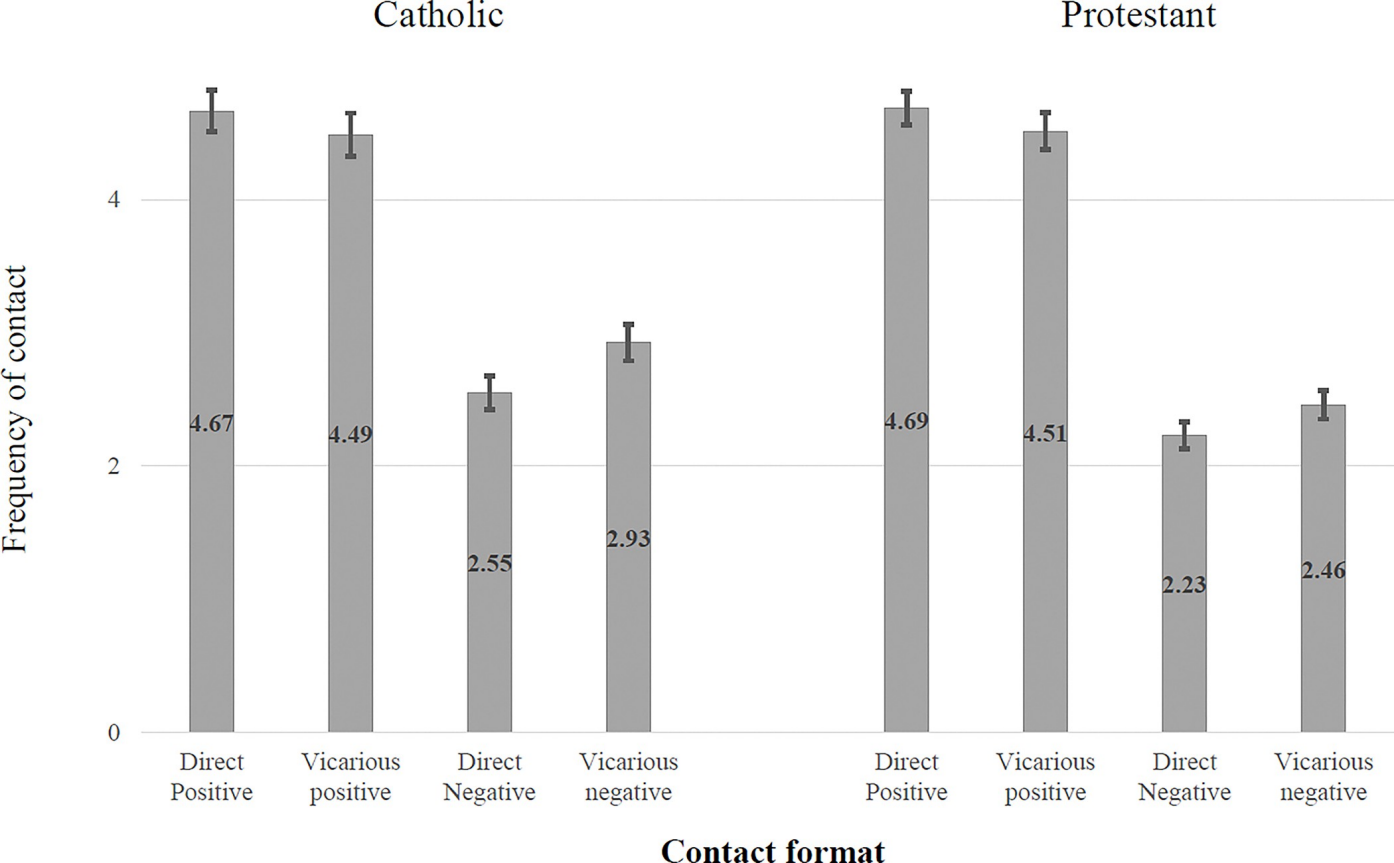
Two-way interaction effects between valence and direct/vicarious contact.

The second analysis looked at the interactive effect of contact valence and directness on contact frequency (Fig 6). In both samples there was significantly more *positive* contact for direct contact than for vicarious contact (Catholic sample t = 4.00, p < .001, Cohen’s d = .11, Protestant sample t = 4.21, p < .001, Cohen’s d = .12) but significantly more *negative* contact for vicarious contact than for direct contact (Catholic sample t = 6.99, p < .001, Cohen’s d = .27, Protestant sample t = 5.64, p < .001, Cohen’s d = .18).

### Relationship between self-reported contact and prejudice

The first analysis repeats the analysis shown in Table 3 for study 1, in examining whether self-reported online and vicarious contact have a significant relationship with prejudice after the effect of face-to-face or direct contact has been removed. Once again, a two-stage hierarchical regression was conducted with the first independent variable being either face-to-face or direct contact and the second independent variable being online or vicarious contact respectively. The analysis was conducted on the Stage 1 data and details of the construction of the prejudice latent variable are given in the supplementary material (see OSM4). Table 6 shows initial R^2^ values and the additional R^2^ that is achieved by adding in the second predictor. When all 8 contact types are included then the regression yields R^2^ = .385 (F(8,438) = 35.96, p< .001), for the Catholic sample and R^2^ = .395 (F(8,558) = 47.19, p< .001) for the Protestant sample.

**Table 6:** Hierarchical regressions of immediacy and directness of contact on prejudice, testing whether adding a contact format less frequently studied (online contact, vicarious contact) improves on a baseline model.

	Sample								
	Catholic					Protestant			
	R^2^	Δ R^2^	F(Δ)	p(Δ)		R^2^	Δ R^2^	F(Δ)	p(Δ)
**F2F / Online**									
1. All F2F contact	0.33	0.33	55.65	< .001		0.31	0.31	63.42	< .001
2. All online contact	0.39	0.06	11.16	< .001		0.40	0.09	21.65	< .001
**Direct / Vicarious**									
1. All Direct contact	0.36	0.36	63.18	< .001		0.38	0.39	89.52	< .001
2. All Vicarious contact	0.39	0.03	5.93	< .001		0.40	0.01	3.36	0.010

Once again, the data show that not only are both contact forms for both samples significant when initially regressing with the more historically researched forms of contact (face-to-face and online), but also that the additional R^2^ achieved by then adding in either online contact or vicarious contact is also statistically significant. For online contact the additional R^2^ (.06 for Catholics, .09 for Protestants) is slightly greater than for Study 1 while for vicarious contact the additional R^2^ (.03 for Catholics, .01 for Protestants) is slightly smaller than for Study 1.

For the remainder of the results section, the analyses are based on data from both stages of the study. All results use only respondents who completed both sections of the study rather than using imputation methods to estimate missing values. To understand the relationship between the contact variables and prejudice, we initially conducted a lagged simple regression analysis on the *two* stages of data using MPlus 8.6 [57]. It consisted of a series of eight separate lagged regressions for each of the eight forms of contact measured at stage one against prejudice measured at stage two, accounting for the value of prejudice at stage one (Table 7). The results can be considered as the lagged equivalent of the bivariate correlations presented in Study 1 (see Table 2 above).

**Table 7:** Lagged individual regression values for contact on prejudice.

	Catholic				Protestant			
Independent variables (Stage 1)	β	S.E.	t	p	β	S.E.	t	p
Positive face-to-face direct	-0.430	0.09	-5.07	< .001	-0.480	0.06	-7.82	< .001
Negative face-to-face direct	0.227	0.08	2.75	0.006	0.291	0.07	4.42	< .001
Positive face-to-face vicarious	-0.414	0.09	-4.70	< .001	-0.518	0.06	-9.11	< .001
Negative face-to-face vicarious	0.410	0.09	4.58	< .001	0.258	0.07	3.72	< .001
Positive online direct	-0.423	0.08	-5.06	< .001	-0.325	0.07	-4.46	< .001
Negative online direct	0.344	0.10	3.30	0.001	0.335	0.06	5.53	< .001
Positive online vicarious	-0.359	0.09	-3.93	< .001	-0.412	0.06	-6.40	< .001
Negative online vicarious	0.287	0.10	2.80	0.005	0.338	0.07	5.00	< .001

As with Study 1, all eight contact formats in both samples were statistically significant and in the expected directions. That is, all four forms of positive contact were associated with reductions in prejudice, and all four forms of negative contact were associated with increases in prejudice. There is no conclusive evidence that negative contact has a stronger relationship with prejudice than positive. Additionally, there are no significant differences to indicate that face-to-face contact has a stronger relationship with prejudice than online contact, or that direct contact has a stronger relationship with prejudice than vicarious contact.

Table 8 below shows the results of running a similar lagged regression of all eight forms of contact on prejudice, but this time with all eight contact variables included at the same time. A combination of the low sample size and the fact that beta values for lagged regressions tend to be lower than for cross-sectional ones [58] means that there are very few significant beta values across the two samples. What can be concluded though is that there is no evidence to indicate that either face-to-face contact is more predictive than online contact, or direct contact more predictive than vicarious contact.

**Table 8:** Lagged multiple regression analysis of all eight contact formats on prejudice.

	Catholic				Protestant			
Independent variables (Stage 1)	β	S.E.	t	p	β	S.E.	t	p
Positive face-to-face direct	-0.049	0.13	-0.372	0.71	-0.166	0.11	-1.505	0.13
Negative face-to-face direct	0.075	0.12	0.641	0.52	0.110	0.09	1.221	0.22
Positive face-to-face vicarious	-0.061	0.14	-0.433	0.67	-0.297	0.11	-2.606	0.01
Negative face-to-face vicarious	0.254	0.11	2.303	0.02	-0.045	0.11	-0.428	0.67
Positive online direct	-0.275	0.16	-1.779	0.08	-0.034	0.11	-0.301	0.76
Negative online direct	0.228	0.15	1.488	0.14	0.128	0.10	1.275	0.20
Positive online vicarious	-0.061	0.14	-0.435	0.66	-0.132	0.12	-1.069	0.29
Negative online vicarious	0.020	0.12	0.159	0.87	0.205	0.10	2.045	0.04

The final analysis examines the paths from contact through the three mediators (Realistic Threat, Symbolic Threat and Intergroup Anxiety) to prejudice. Because of the number of variables involved in the SEM, the sample size is not sufficiently robust to run full SEMs on the sectarian subgroups [59] and is marginal for running the analysis in total. Instead, a full mediated path analysis was run with MPlus 8.6 using average scores for the mediators and prejudice rather than a full SEM to create the latent variables. We also focused on the four forms of contact most central to the current paper, namely positive and negative face-to-face contact and positive and negative online contact.

Prior to running the analysis, the statement reliabilities were checked. For the four variables (prejudice, anxiety, realistic threat and symbolic threat), the McDonald’s ω figures are shown in Table 9, which shows that all are acceptably high. Although the analysis did not create the variables, we did test metric invariance of the underlying latent variable constructs across the two stages [60]. In all cases the χ2 difference between the constrained and the unconstrained CFA were not significant (Table 9), and so invariance can be assumed.

**Table 9:** Reliability and invariance analyses for latent variables.

	Prejudice	Anxiety	Realistic Threat	Symbolic Threat
McDonald’s ω stage 1	0.916	0.856	0.898	0.843
McDonald’s ω stage 2	0.919	0.887	0.888	0.861
χ2 difference with/without loading constraints	9.37	2.79	5.11	4.27
df	6	5	4	4
p	0.154	0.732	0.277	0.371

The analysis consisted of a path analysis following the path from the contact variables at stage one to the mediators at stage two to prejudice at stage two [61,62]. The analysis was run using MPlus8.6.

**Fig 7:**
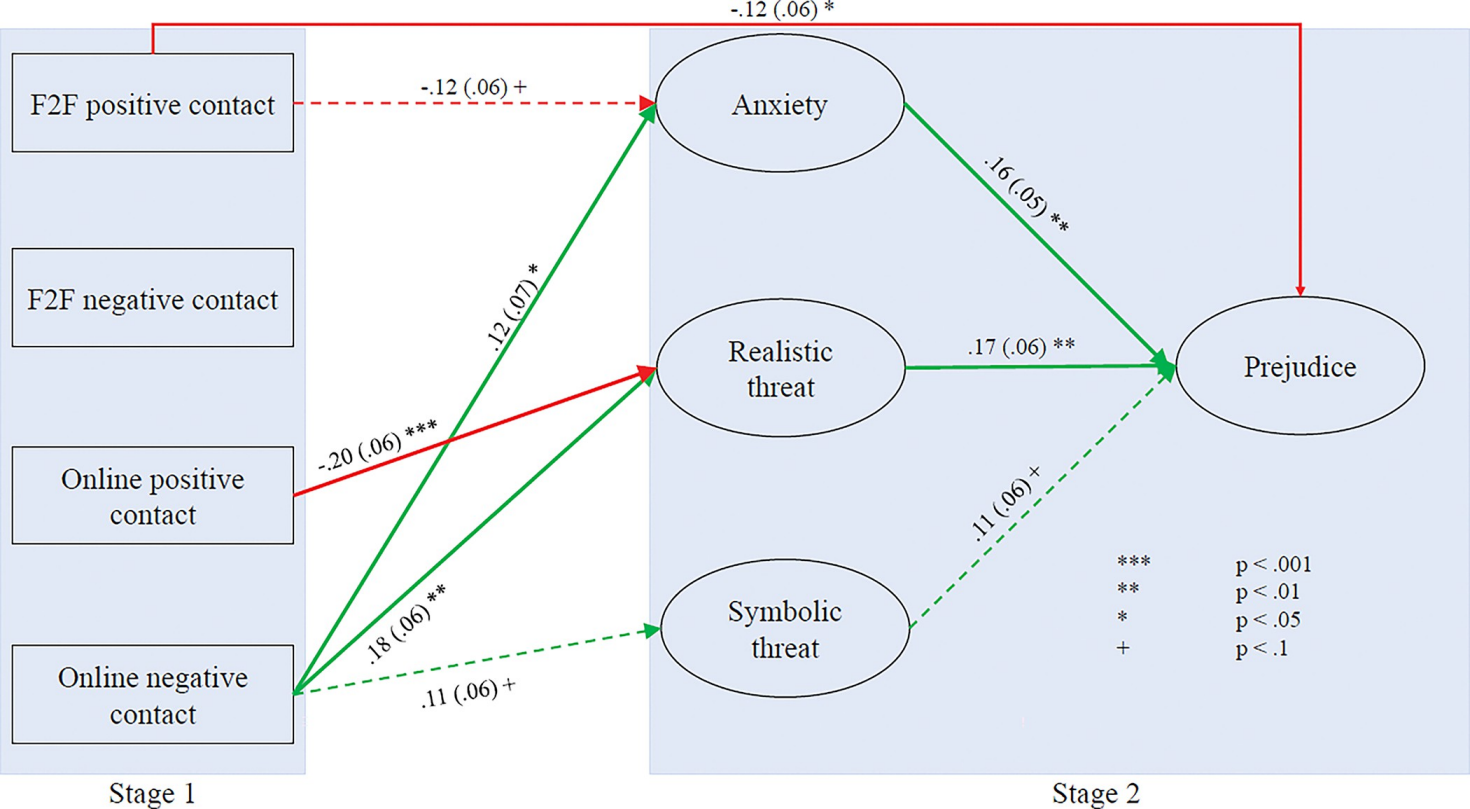
Mediation model of the effects of face-to-face and online contact on prejudice.

The results of the path analysis are shown in Fig 7. The figure displays all the significant paths between the variables, excluding the autoregressive path between prejudice at stage one and prejudice at stage two. The model fit statistics (RMSE = .045, CFI = .993, TLI = .977) were good [63,64]. Data for all paths are shown in the supplementary material (see OSM5), together with the bootstrap estimations of confidence intervals.

Due to the smaller than expected sample size, statistically significant paths at the 1 in 10 level have been shown provided that the standardized beta coefficient is greater than 0.1. There are significant paths from all mediators to prejudice. The only paths from face-to-face contact are from positive face-to-face contact. There is a marginally significant path to anxiety as well as a direct significant path to prejudice. Conversely, there are significant paths from negative online contact to all three of the mediators together with a significant path from positive online contact to realistic threat. Overall, the results indicate no evidence to suggest that online contact works through different mediators, and certainly no evidence to support any claim to primacy for face-to-face contact over online in terms of the relationship with prejudice.

## Discussion

Study 2 replicated, refined, and extended the results of Study 1. First, as with Study 1, positive contact was experienced more frequently by Catholic and Protestant respondents than negative contact in both online and face-to-face contexts. This simple pattern, however, was qualified by more nuanced interactional patterns, particularly with respect to contact valence. As in Study 1, positive contact occurred more often in face-to-face than in online contexts for both Protestant and Catholic respondents. However, face-to-face negative contact did not occur more frequently than negative contact online for Protestants. Moreover, negative online contact was actually experienced *more frequently* by Catholics than negative face-to-face contact. Finally, it is also worth noting that vicarious negative contact experiences, though relatively neglected in the social psychology literature, were more common than direct negative contact experiences for both Catholic and Protestant respondents.

Second, although positive face-to-face contact remained the strongest predictor of sectarian prejudice in Study 2, online and negative contact were also significant predictors and explained unique variance in terms of levels of prejudice. For example, as captured in Fig 7, online positive and negative contact were both significantly associated with sectarian attitudes, even when controlling for their face-to-face equivalents. As in Study 1, then, this pattern of findings indicates that virtual forms of contact are not necessarily secondary in terms of their impact on prejudice or mere ‘stepping stones’ to promote real interactions. They are important in their own right.

Third, Study 2 also attempted to explore some variables that potentially mediate the relationship between different forms of contact and sectarian prejudice. For face-to-face contact, there is only one partially mediated path from contact to prejudice (positive face-to-face through anxiety). Conversely, for online contact, the relationship between prejudice and negative online contact is partially mediated by all three mediators. This is similar to other work in this area [5,17,48,54].

The fact that online but not off-line experiences of negative contact seem to increase sectarian prejudice via their impact on intergroup anxiety is perhaps puzzling. On the one hand, by definition, such contact occurs relatively anonymously and at distance and thus, in theory, it should insulate participants from the kinds of direct interactional threats that heighten intergroup anxiety. As such, one might expect face-to-face negative contact to be more interactionally challenging than contact online and thus to have a greater impact on intergroup attitudes. On the other hand, we would conjecture that online negative contact experiences may be *qualitatively* more severe in their negativity than face-to-face experiences. That is, factors such as anonymity may increase the likelihood of extreme and anxiety-provoking exchanges in virtual contexts, a theme that resonates with work on cyber bullying [65,66]. Another hypothesis from a reviewer was that an individual’s ability to develop mechanisms to cope with negative contact might vary depending on whether the contact was online or face-to-face. The design of the present research does not allow us to resolve this issue, but we highlight it below as a question that future researchers might address.

## General discussion

The present research was designed to systematically compare the frequency with which individuals experience varying forms of online and face-to-face contact and to explore the relationship between these contact experiences and self-reported prejudice. Unlike most previous research [21], our studies focused on general and everyday forms of online contact rather than contact unfolding under controlled conditions or as part of an intervention to improve intergroup relations. Our overarching aim was to interrogate the assumption—implicit in much of the social psychological literature on the contact hypothesis—that positive face-to-face contact is of primary significance in understanding and transforming intergroup attitudes. In a world where communication is increasingly accomplished via virtual platforms and technologies, this is an important question and, in our view, will become increasingly important in the future.

Our main finding is simple and clearcut. Across all four groups that we surveyed in Studies 1 and 2, namely Black and White populations in the UK and Catholics and Protestants in Northern Ireland, positive face-to-face contact was: (a) experienced more frequently than any other form of contact and (b) had consistently the strongest association with racial (Study 1) and sectarian (Study 2) prejudice. In sum, our research provided support for the traditional contact hypothesis [2,3] and confirmed the enduring significance of face-to-face interaction in shaping intergroup attitudes, even in a social world where virtual forms of communication are increasingly prevalent.

Our research also provided evidence that qualified and complicated this simple picture, however. First, although positive face-to-face contact was the strongest predictor of intergroup attitudes, other forms of contact were also significant predictors within the multivariate models presented in Studies 1 and 2. Adding these forms of contact as predictors increased the overall predictive strength of our models and suggested, for example, that online and indirect contact experiences explain variations in intergroup attitudes beyond the effects of face-to-face interactions.

Second and related, we found that negative contact (but not positive) experiences occurred as frequently online as they did face-to-face, and in some cases more frequently (e.g., for Catholic respondents in Study 2). Moreover, in both our studies, online negative contact experiences had as strong an association with prejudice as face-to-face negative contact experiences, and in the case of Black participants in Study 1 a stronger association. That is, as expected, for Black participants, positive contact with White people was generally associated with lowered prejudice levels and negative contact with increased prejudice levels [25]. However, in addition, while positive contact had the strongest relationship to lower prejudice when experienced *face-to-face*, negative contact–both direct and vicarious—had a stronger relationship to prejudice accentuation when experienced *online*. This pattern may reflect the fact that historically disadvantaged racial groups are more likely to be targeted for racist abuse in online environments, where anonymity and lack of direct accountability make the overt expressions of prejudice more likely. What is clear, however, is that if the contact hypothesis were to be proposed today it is inconceivable that it would not have included a wider definition of contact, particular regarding intergroup interaction online.

Third, Study 2 sought to explore some affective mediators of online and face-to-face contact, focusing on realistic threat, symbolic threat and intergroup anxiety [48,54]. Our results indicated that for negative forms of contact, online and not face-to-face contact influenced prejudice through the three mediators. As mentioned above in our discussion of Study 2, this pattern may reflect features of the content of negative online and face-to-face contact, though this was not directly explored in the present research. In our view, it is plausible that negative online contact between Catholics and Protestants in Northern Ireland may feature more extreme expressions of sectarianism, thus cueing, for example, heightened intergroup anxiety. This is a potential avenue for future research.

## Limitations and future directions

In the present research, contact was measured in a way that enabled a simple, efficient and generic comparison between different forms of online and face-to-face contact. However, this approach has obvious limitations. Notably, by using a single rather than multi-dimensional measure of contact frequency, it neglects the contextual richness and variability of contact experience. Future research would benefit from using more nuanced measures of contact to better understand whether there are qualitative as well as quantitative differences between different forms of contact experiences across different face-to-face and online contexts.

This is especially relevant for online negative contact. Study 1 demonstrated a reduction in prejudice linked to negative online contact rather than the expected increase. As discussed earlier, other studies [50,51] have produced similar results, particularly linked to extended or parasocial contact. It would be valuable therefore to conduct more nuanced work on negative online extended contact to assess how participants are understanding this form of contact and why it is negatively related to prejudice. This might fruitfully be conducted in the context of a simple sender-receiver communication theory model [67] in order to establish whether the effect occurs as a result of a difference in the contact *itself* when it is online compared to face-to-face, or a difference in the *reaction* to the contact from the observer.

Additionally, the measurement approach we adopted did not differentiate between asynchronous social media contact on platforms such as Facebook or Twitter and synchronous contact accomplished through technologies such as Zoom and Skype. This raises questions regarding the interaction between different forms of online communication and contact valence. It has been hypothesised [33], for instance, that asynchronous contact is likely to lead to more disinhibition than synchronous contact. As such, asynchronous online contact may have greater potential to become negative and damaging [9]. It is also possible that online contact with outgroup members with whom an individual has a pre-existing, face-to-face relationship is quite different from online contact with outgroup members with whom an individual has had no prior interactions. Similarly, online vicarious contact between friends of friends on Facebook or Twitter might be different to online vicarious contact consisting of, for example, exposure to intergroup interactions displayed on TV, in films or advertisements featuring intergroup encounters [68]. These are all issues that future research might explore.

It is worth noting in conclusion that the present research was conducted during the Covid 19 pandemic, at a time when interpersonal and intergroup communication profoundly altered and online communication dramatically increased. This context may well have shaped how our participants responded to survey questions about their experiences of different forms of contact, and our results must be interpreted with this in mind. Moreover, it is likely that the pandemic will further accelerate the future frequency of online communications in contexts such as the workplace. It is essential contact researchers remain alive to the evolving nature of such communications and to their consequences both for intergroup prejudice and for wider patterns of intergroup and related policy attitudes.

## Supporting information

S1 File Supplementary material 1 –Study questionnaires study 1 –UK BLM. (PDF)

S2 File Supplementary material 1 –Study questionnaires study 2 –Northern Ireland. (PDF)

S3 File Supplementary material 2 –Sample structure study 1. (PDF)

S4 File Supplementary material 2 –Sample structure study 2. (PDF)

S5 File Supplementary material 3 –Correlations of contact by format. (PDF)

S6 File Supplementary material 4 –Latent variable construction study 1. (PDF)

S7 File Supplementary material 4 –Latent variable construction study 1 study 2. (PDF)

S8 File Supplementary material 5 –Regression summary tables study 1(variables selected by forward stepwise procedure). (PDF)

S9 File Supplementary material 6 –Full sem path tables study 2. (PDF)
