# Supplementary material for: The contact hypothesis and the virtual revolution: Does face-to-face interaction remain central to improving intergroup relations?
Source: PLoS One. 2023 Dec 8;18(12):e0292831. doi: 10.1371/journal.pone.0292831 (PMC10707701; doi:10.1371/journal.pone.0292831)
Supplement: S1 File — (PDF) [file pone.0292831.s001.pdf]

SM1 Study one Questionnaire

Q Which of the descriptions below best describes you?

White British

Asian British

Black / Afro-Caribbean British

British citizen of other European origin

British citizen of North / South America

Other

Rather not say

Q On average, how often do you have positive / good FACE-TO-FACE contact with (outgroup)?

Q On average, how often do you have negative / bad FACE-TO-FACE contact with (outgroup)?

Q On average, how often do you observe positive / good FACE-TO-FACE contact between white and black people?

Q On average, how often do you observe negative / bad FACE-TO-FACE contact between white and black people?

Q On average, how often do you have positive / good ONLINE contact with (outgroup)?

Q On average, how often do you have negative / bad ONLINE contact with (outgroup)?

Q2 On average, how often do you observe positive / good ONLINE contact between black and white people?

Q On average, how often do you observe negative / bad ONLINE contact between black and white people?

Scale:

Never

Not at all frequently

Not very frequently

Quite frequently

Reasonably frequently

Very frequently

Extremely Frequently

Q I am now going to show you some statements that people have made. For each one I'd like you to say how much you agree or disagree with the statement. Again, there are no right or wrong answers, it is your opinion that we are interested in.

(Outgroup) have more political power in Britain than they should

(Outgroup) have more economic power in Britain than they should

(Outgroup) get preferential access to better jobs in Britain than they should

(Outgroup) are more responsible for the level of crime than ours

The police force provides greater support to (outgroup) than they should

Scale:

Strongly Disagree

Disagree

Neither agree nor disagree

Agree

Strongly Agree

Q And here are some more statements that people have made. Again, for each one I'd like you to say how much you agree or disagree with the statement. Again, there are no right or wrong answers, it is your opinion that we are interested in.

(Outgroup) have different family values than (ingroup)

(Outgroup) have a different attitude to work than (ingroup)

(Outgroup)'s beliefs are incompatible with ours

(Outgroup) is changing our way of life

(Outgroup) represents a threat to our traditional values

Scale:

Strongly Disagree

Disagree

Neither agree nor disagree

Agree

Strongly Agree

Q Now, can you tell me, overall, how you feel about (outgroup) in general? Again, there are no right or wrong answers, it is your opinions that we are interested in.

Extremely unfavourable

Very unfavourable

Quite unfavourable

Fairly unfavourable

Slightly unfavourable

Neither favourable nor unfavourable

Slightly favourable

Fairly favourable

Quite favourable

Very favourable

Extremely favourable

Q And now, on the scales below, can you tell me for each one how you would describe your feeling towards (outgroup) in general? Again, there are no right or wrong answers, it is your opinions that we are interested in.

ROTATE ORDER OF STATEMENTS

Warm → Cold

Negative → Positive

Friendly → Hostile

Suspicious → Trusting

Respect → Contempt

Admiration → Disgust
