# Supplementary material for: The contact hypothesis and the virtual revolution: Does face-to-face interaction remain central to improving intergroup relations?
Source: PLoS One. 2023 Dec 8;18(12):e0292831. doi: 10.1371/journal.pone.0292831 (PMC10707701; doi:10.1371/journal.pone.0292831)
Supplement: S2 File — (PDF) [file pone.0292831.s002.pdf]

SM1 Study 2 Questionnaire

Q Firstly, which of the descriptions below best describes your religious affiliations?

Catholic

Protestant

Other (including no religious affiliation)

Rather not say

Q On average, how often do you have positive / good FACE-TO-FACE contact with (outgroup)?

Q On average, how often do you have negative / bad FACE-TO-FACE contact with (outgroup)?

Q On average, how often do you observe positive / good FACE-TO-FACE contact between Catholics and Protestants?

Q On average, how often do you observe negative / bad FACE-TO-FACE contact between Catholics and Protestants?

Q On average, how often do you have positive / good ONLINE contact with (outgroup)?

Q On average, how often do you have negative / bad ONLINE contact with (outgroup)?

Q2 On average, how often do you observe positive / good ONLINE contact between Catholics and Protestants?

Q On average, how often do you observe negative / bad ONLINE contact between Catholics and Protestants?

Scale:

Never

Not at all frequently

Not very frequently

Quite frequently

Reasonably frequently

Very frequently

Extremely Frequently

Q We would like to ask you now about how you would feel in Northern Ireland mixing socially with complete strangers who were members of the other community. It doesn't matter whether you personally have very little or no contact with members of the other community, please try to imagine how you would feel. If you were the only person from your own community and you

found yourself with a group of people from the other community. How would you feel compared to an occasion where you found yourself with people of only your community?

Rotate order of Descriptors

Happy

Awkward

Self-conscious

Confident

Relaxed

Defensive

Scale:

Not at all

A little

Some

Quite

Extremely

Q I am now going to show you some statements that people have made. For each one I'd like you to say how much you agree or disagree with the statement. Again, there are no right or wrong answers, it is your opinion that we are interested in.

The other community has more political power in Northern Ireland than they should

The other community has more economic power in Northern Ireland than they should

The other community gets preferential access to better jobs in Northern Ireland than they should

The other community is more responsible for the level of crime than ours

The police force provides greater support to the other community than it should

Scale:

Strongly Disagree

Disagree

Neither agree nor disagree

Agree

Strongly Agree

Q And here are some more statements that people have made. Again, for each one I'd like you to say how much you agree or disagree with the statement. Again, there are no right or wrong answers, it is your opinion that we are interested in.

The other community has different family values than ours

The other community has a different attitude to work than ours

The other community's religion is incompatible with ours

The other community is changing our way of life

The other community represents a threat to our traditional values

Scale:

Strongly Disagree

Disagree

Neither agree nor disagree

Agree

Strongly Agree

Q As you might be aware The Northern Irish government plans to significantly increase the amount of integration in schooling creating more mixing of Protestant and Catholic children. You are going to see some statements that people have made about this. Can you tell me how much you agree or disagree with each statement. Again, there are no right or wrong answers, it is your opinions that we are interested in.

RANDOMISE ORDER OF STATEMENTS

I support government proposals to increase integration in schools

It is too soon to significantly increase the level of integration in schools.

Segregation in schooling is a thing of the past and the government is right to target their change to greater integration.

Scale:

Strongly disagree

Disagree

Neither agree nor disagree

Agree

Strongly agree

Q Also, the Northern Irish government is planning to take down the interface barriers or "peace walls" across Belfast. Below are some statements that people have made about this. Can you tell me how much you agree or disagree with each statement. Again, there are no right or wrong answers, it is your opinions that we are interested in.

RANDOMISE ORDER OF STATEMENTS

I support government proposals to remove interface barriers

It is too soon to remove interface barriers in Belfast

Interface barriers are a thing of the past and the government is right to target their removal

Scale:

Strongly disagree

Disagree

Neither agree nor disagree

Agree

Strongly agree

Q Now, can you tell me, overall, how you feel about (outgroup) in general? Again, there are no right or wrong answers, it is your opinions that we are interested in.

Extremely unfavourable

Very unfavourable

Quite unfavourable

Fairly unfavourable

Slightly unfavourable

Neither favourable nor unfavourable

Slightly favourable

Fairly favourable

Quite favourable

Very favourable

Extremely favourable

Q And now, on the scales below, can you tell me for each one how you would describe your feeling towards (outgroup) in general? Again, there are no right or wrong answers, it is your opinions that we are interested in.

ROTATE ORDER OF STATEMENTS

Warm → Cold

Negative → Positive

Friendly → Hostile

Suspicious → Trusting

Respect → Contempt

Admiration → Disgust
