# Supplementary material for: The contact hypothesis and the virtual revolution: Does face-to-face interaction remain central to improving intergroup relations?
Source: PLoS One. 2023 Dec 8;18(12):e0292831. doi: 10.1371/journal.pone.0292831 (PMC10707701; doi:10.1371/journal.pone.0292831)
Supplement: S3 File — (PDF) [file pone.0292831.s003.pdf]

SM2 Study 1 Sample

The questionnaire forced answers to all questions so there was no issue in either sample with missing data. Data were checked and all respondents showing no discrimination across all the statements and taking less than three minutes to complete the questionnaire were removed. This reduced the sample size from 1084 to 1030. Data were collected in September 2020.

Sample - Study 1

	Total		White British		Black / Afro-Caribbean British	
	Count	%	Count	%	Count	%
	1030	100%	529	100%	501	100%
SAMPLE						
White British	529	51.4%	529	100.0%	0	0.0%
Black / Afro-Caribbean British	501	48.6%	0	0.0%	501	100.0%
AGE						
18-24	194	18.8%	75	14.2%	119	23.8%
25-34	227	22.0%	101	19.1%	126	25.1%
35-44	191	18.5%	95	18.0%	96	19.2%
45-54	179	17.4%	89	16.8%	90	18.0%
55-64	134	13.0%	90	17.0%	44	8.8%
65-74	82	8.0%	65	12.3%	17	3.4%
75+	16	1.6%	14	2.6%	2	0.4%
Prefer not to say	7	0.7%	0	0.0%	7	1.4%
GENDER						
Female	560	54.4%	275	52.0%	285	56.9%
Male	470	45.6%	254	48.0%	216	43.1%
