# Supplementary material for: The contact hypothesis and the virtual revolution: Does face-to-face interaction remain central to improving intergroup relations?
Source: PLoS One. 2023 Dec 8;18(12):e0292831. doi: 10.1371/journal.pone.0292831 (PMC10707701; doi:10.1371/journal.pone.0292831)
Supplement: S4 File — (PDF) [file pone.0292831.s004.pdf]

SM2 Study 2 Sample

As for study 1, the questionnaire forced answers to all questions so there was no issue in either sample with missing data. Data were checked and all respondents showing no discrimination across all the statements and taking less than three minutes to complete the questionnaire were removed. This reduced the sample size at stage 1 from 1073 to 1014, and the sample size at stage 2 from 268 to 249. Data for stage 1 were collected in November 2019. Data for stage 2 were collected in March / April 2020.

Sample - Study 2 – Stage 1

	Total		Catholic		Protestant	
	Count	%	Count	%	Count	%
	1014	100%	447	100%	567	100%
SAMPLE						
Catholic	447	44.1%	447	100.0%	0	0.0%
Protestant	567	55.9%	0	0.0%	567	100.0%
AGE						
18-24	130	12.8%	65	14.5%	65	11.5%
25-34	269	26.5%	141	31.5%	128	22.6%
35-44	238	23.5%	112	25.1%	126	22.2%
45-54	190	18.7%	69	15.4%	121	21.3%
55-64	118	11.6%	41	9.2%	77	13.6%
65-74	61	6.0%	17	3.8%	44	7.8%
75+	6	0.6%	0	0.0%	6	1.1%
Prefer not to say	2	0.2%	2	0.4%	0	0.0%
GENDER						
Female	636	62.7%	289	64.7%	347	61.2%
Male	376	37.1%	158	35.3%	218	38.4%
Prefer not to say	2	0.2%	0	0.0%	2	0.4%

Sample - Study 2 – Stage 2

	Total		Catholic		Protestant	
	Count	%	Count	%	Count	%
	249	100%	97	100%	152	100%
SAMPLE						
Catholic	97	39.0%	447	100.0%	0	0.0%
Protestant	152	61.0%	0	0.0%	567	100.0%
AGE						
18-24	14	5.6%	6	6.2%	8	5.3%
25-34	51	20.5%	24	24.7%	27	17.8%
35-44	60	24.1%	28	28.9%	32	21.1%
45-54	55	22.1%	20	20.6%	35	23.0%
55-64	46	18.5%	14	14.4%	32	21.1%
65-74	21	8.4%	5	5.2%	16	10.5%
75+	2	0.8%	0	0.0%	2	1.3%
GENDER		0.0%				
Female	146	58.6%	61	62.9%	85	55.9%
Male	103	41.4%	36	37.1%	67	44.1%

Comparison of stage 1 only sample (N=350) and both stage sample (N=97) Catholic Sample

	Stage 1 & 2		Stage 1 only				
	**M**	**S.E.**	**M**	**S.E.**	**Difference**	**t**	**p**
Positive face-to-face direct	5.00	0.173	5.06	0.098	-0.06	-0.275	0.784
Negative face-to-face direct	2.52	0.157	2.63	0.088	-0.12	-0.625	0.532
Positive face-to-face vicarious	4.96	0.170	4.81	0.099	0.15	0.710	0.478
Negative face-to-face vicarious	2.59	0.156	2.65	0.082	-0.07	-0.380	0.704
Positive online direct	4.09	0.188	4.34	0.101	-0.25	-1.144	0.253
Negative online direct	2.23	0.152	2.56	0.088	-0.33	-1.802	0.072
Positive online vicarious	3.95	0.177	4.19	0.101	-0.24	-1.108	0.268
Negative online vicarious	3.04	0.173	3.27	0.094	-0.22	-1.121	0.263
Anxiety	2.62	0.107	2.55	0.052	0.07	0.588	0.557
Realistic Threat	3.30	0.092	3.08	0.057	0.21	1.784	0.075
Symboic Threat	2.44	0.098	2.44	0.054	0.01	0.051	0.959
Prejudice	2.39	0.102	2.50	0.050	-0.12	-1.068	0.286

Comparison of stage 1 only sample (N=415) and both stage sample (N=152) Protestant Sample

	Stage 1 & 2		Stage 1 only				
	**M**	**S.E.**	**M**	**S.E.**	**Difference**	**t**	**p**
Positive face-to-face direct	5.15	0.130	5.10	0.080	0.05	0.309	0.757
Negative face-to-face direct	2.28	0.122	2.24	0.070	0.04	0.287	0.775
Positive face-to-face vicarious	4.82	0.137	4.83	0.084	-0.01	-0.040	0.968
Negative face-to-face vicarious	2.49	0.115	2.39	0.066	0.10	0.763	0.446
Positive online direct	4.24	0.153	4.27	0.091	-0.04	-0.215	0.830
Negative online direct	2.13	0.118	2.23	0.068	-0.10	-0.749	0.454
Positive online vicarious	4.19	0.149	4.20	0.085	-0.01	-0.084	0.933
Negative online vicarious	2.26	0.107	2.60	0.074	-0.34	-2.454	0.014
Anxiety	2.36	0.077	2.37	0.048	-0.01	-0.070	0.945
Realistic Threat	2.55	0.081	2.61	0.047	-0.06	-0.697	0.486
Symboic Threat	2.63	0.079	2.55	0.044	0.07	0.855	0.393
Prejudice	2.33	0.069	2.42	0.042	-0.08	-1.041	0.298

Contact Comparison - Study 1 / Study 2 – Catholic Sample

	**Stage 1**		**Stage 2**				
**Contact**	**m**	**SE**	**m**	**SE**	**diff**	**t (diff)**	**p**
Positive face-to-face direct	5.04	0.09	4.95	0.16	0.10	0.49	0.627
Negative face-to-face direct	2.61	0.08	2.59	0.16	0.02	0.10	0.917
Positive face-to-face vicarious	4.84	0.09	4.82	0.16	0.02	0.09	0.925
Negative face-to-face vicarious	2.64	0.07	2.69	0.15	-0.05	-0.30	0.765
Positive online direct	4.29	0.09	4.36	0.18	-0.07	-0.36	0.721
Negative online direct	2.49	0.08	2.40	0.15	0.09	0.48	0.633
Positive online vicarious	4.13	0.09	4.28	0.17	-0.14	-0.70	0.484
Negative online vicarious	3.22	0.08	2.91	0.16	0.31	1.60	0.109

Contact Comparison - Study 1 / Study 2 – Protestant Sample

	**Stage 1**		**Stage 2**				
**Contact**	**m**	**SE**	**m**	**SE**	**diff**	**t (diff)**	**p**
Positive face-to-face direct	5.12	0.07	4.92	0.13	0.20	1.31	0.189
Negative face-to-face direct	2.25	0.06	2.20	0.11	0.06	0.43	0.664
Positive face-to-face vicarious	4.83	0.07	4.88	0.13	-0.05	-0.31	0.757
Negative face-to-face vicarious	2.41	0.06	2.33	0.10	0.09	0.70	0.485
Positive online direct	4.26	0.08	4.38	0.15	-0.12	-0.69	0.492
Negative online direct	2.20	0.06	2.09	0.11	0.11	0.88	0.380
Positive online vicarious	4.20	0.07	4.25	0.15	-0.05	-0.30	0.762
Negative online vicarious	2.50	0.06	2.38	0.11	0.13	0.99	0.324
