# Supplementary material for: The contact hypothesis and the virtual revolution: Does face-to-face interaction remain central to improving intergroup relations?
Source: PLoS One. 2023 Dec 8;18(12):e0292831. doi: 10.1371/journal.pone.0292831 (PMC10707701; doi:10.1371/journal.pone.0292831)
Supplement: S5 File — (PDF) [file pone.0292831.s005.pdf]

SM3 Correlations of Contact Formats

Study 1. Black Sample (n=501)

	Mean	SD	1	2	3	4	5	6	7
Positive face-to-face direct (1)	4.87	1.404							
Negative face-to-face direct (2)	3.13	1.458	-0.020						
Positive face-to-face vicarious (3)	4.58	1.372	.508***	.113*					
Negative face-to-face vicarious (4)	3.51	1.538	-0.012	.615***	.135**				
Positive online direct (5)	4.47	1.496	.545***	0.048	.483***	0.059			
Negative online direct (6)	3.08	1.618	-0.050	.641***	.106*	.548***	0.069		
Positive online vicarious (7)	4.32	1.489	.380***	.136**	.536***	.120**	.567***	.178***	
Negative online vicarious (8)	3.70	1.646	0.086	.537***	.152***	.574***	0.048	.581***	.134**

All measures have a range from 1 to 7
*** p .001, ** p < .01, * p < .05

Study 1. White sample (n=529)

	Mean	SD	1	2	3	4	5	6	7
Positive face-to-face direct (1)	4.25	1.737							
Negative face-to-face direct (2)	2.10	1.432	0.052						
Positive face-to-face vicarious (3)	4.35	1.575	.707***	0.030					
Negative face-to-face vicarious (4)	2.66	1.445	.137**	.574***	.139**				
Positive online direct (5)	3.82	1.820	.634***	.181***	.583***	.194***			
Negative online direct (6)	1.97	1.406	.096*	.686***	0.055	.571***	.239***		
Positive online vicarious (7)	3.86	1.719	.527***	.150***	.600***	.184***	.650***	.171***	
Negative online vicarious (8)	2.79	1.560	.107*	.488***	.144***	.583***	.268***	.547***	.253***

All measures have a range from 1 to 7
*** p .001, ** p < .01, * p < .05

Study 2. Catholic sample (n=447)

	Mean	SD	1	2	3	4	5	6	7
Positive face-to-face direct (1)	5.04	1.810							
Negative face-to-face direct (2)	2.61	1.616	-.122**						
Positive face-to-face vicarious (3)	4.84	1.809	.734***	-0.079					
Negative face-to-face vicarious (4)	2.64	1.528	-0.074	.503***	-.106*				
Positive online direct (5)	4.29	1.884	.618***	0.004	.625***	-0.040			
Negative online direct (6)	2.49	1.615	-.122**	.513***	-.094*	.553***	-0.019		
Positive online vicarious (7)	4.13	1.867	.631***	-0.056	.605***	-0.017	.720***	-0.017	
Negative online vicarious (8)	3.22	1.746	0.007	.361***	0.033501	.474***	-0.005	.573***	0.040

All measures have a range from 1 to 7
*** p .001, ** p < .01, * p < .05

Study 2. Protestant sample (n=567)

	Mean	SD	1	2	3	4	5	6	7
Positive face-to-face direct (1)	5.12	1.627							
Negative face-to-face direct (2)	2.25	1.453	-.128**						
Positive face-to-face vicarious (3)	4.83	1.708	.704***	-0.034					
Negative face-to-face vicarious (4)	2.41	1.367	-.164***	.535***	-.099*				
Positive online direct (5)	4.26	1.855	.507***	-0.043	.491***	-0.008			
Negative online direct (6)	2.20	1.404	-.160***	.554***	-.138**	.560***	-0.026		
Positive online vicarious (7)	4.20	1.760	.516***	-0.036	.565***	-0.049	.705***	-0.022	
Negative online vicarious (8)	2.50	1.462	-.094*	.465***	-.092*	.581***	-0.007	.642***	0.029

All measures have a range from 1 to 7
*** p .001, ** p < .01, * p < .05
