# Supplementary material for: The contact hypothesis and the virtual revolution: Does face-to-face interaction remain central to improving intergroup relations?
Source: PLoS One. 2023 Dec 8;18(12):e0292831. doi: 10.1371/journal.pone.0292831 (PMC10707701; doi:10.1371/journal.pone.0292831)
Supplement: S6 File — (PDF) [file pone.0292831.s006.pdf]

SM4 Study One Latent Variable Construction

The prejudice variable was derived from the Likert questions and feelings thermometer using SPSS v.27. The Likert questions were scaled as 1-5 and negative statements were reverse scored. The feelings thermometer was rescaled so that the scores ranged from 1-5 rather than 1-11. McDonald's ω scores were .85 for the black sample and .91 for the white sample. In both cases, neither figure could be improved by removing any of the variables. While these figures can be regarded as very good, an additional analysis was conducted which measured the average interitem correlations. The results were 0.44 for the black sample and 0.60 for the white sample. These are comfortably above the recommended minimum value of 0.15 .

To check unidimensionality, an exploratory factor analysis (EFA) was run on the seven variables. For both samples the second factor has an eigenvalue less than one, indicating that the construct is unidimensional. Additionally, all the exogenous variables have loadings over 0.5 which eliminates the need to reduce the number of exogenous variables used (Field, 2009).

Exploratory factor analysis for latent prejudice variable

	Black sample	White sample
Factor 1 Eigenvalue	3.664	4.617
Factor 2 Eigenvalue	0.860	0.673
Min. factor loading	0.588	0.696
Max. factor loading	0.810	0.869

Finally, a CFA was run on the 7 statements using MPlus 8.6. The model fit statistics were: χ^2 = 18.21, df = 12, p = .110, RMSEA = .032, CFI = .995, TLI = .991, SRMR = .019 for the black sample and χ^2 = 4.01, df = 7, p = .779, RMSEA = .057, CFI = .993, TLI = .985, SRMR = .021 for the white sample
