# Supplementary material for: The contact hypothesis and the virtual revolution: Does face-to-face interaction remain central to improving intergroup relations?
Source: PLoS One. 2023 Dec 8;18(12):e0292831. doi: 10.1371/journal.pone.0292831 (PMC10707701; doi:10.1371/journal.pone.0292831)
Supplement: S7 File — (PDF) [file pone.0292831.s007.pdf]

SM4 Study 2 Latent Variable Construction

The prejudice latent variables were again derived using SPSS v.27. As before, to create the prejudice variable the Likert questions were scaled as 1-5 and negative statements were reverse scored. The feelings thermometer was rescaled so that the scores ranged from 1-5 rather than 1-11. McDonald's ω scores were .918 for the Catholic sample and .915 for the Protestant sample. In both cases these figures are good and neither figure could be improved by removing any of the variables. The average interitem correlations were 0.61 for Catholics and 0.59 for Protestants, well above the recommended minimum value of 0.15.

An exploratory factor analysis (EFA) was run on the seven variables. For both samples the latent variable was unidimensional as the second factor had an eigenvalue less than one. All the exogenous variables had loadings over 0.5 so that none of them need to be removed.

Exploratory factor analysis for latent prejudice variable

	Catholic	Protestant
Factor 1 Eigenvalue	4.660	4.582
Factor 2 Eigenvalue	0.618	0.682
Max. factor loading	0.882	0.865
Min. factor loading	0.678	0.597

Finally, a CFA was run on the 7 statements using MPlus 8.6. The model fit statistics were: $\chi^2 = 14.27$, df = 10, p = .161, RMSEA = .031, CFI = .998, TLI = .996, SRMR = .014 for the Catholic sample and $\chi^2 = 19.98$, df = 7, p = .006, RMSEA = .057, CFI = .995, TLI = .985, SRMR = .014 for the Protestant sample
