# Supplementary material for: The contact hypothesis and the virtual revolution: Does face-to-face interaction remain central to improving intergroup relations?
Source: PLoS One. 2023 Dec 8;18(12):e0292831. doi: 10.1371/journal.pone.0292831 (PMC10707701; doi:10.1371/journal.pone.0292831)
Supplement: S8 File — (PDF) [file pone.0292831.s008.pdf]

SM5 Study one Regression Summary tables

BLACK SAMPLE

	β	SE(β)	t	p	Cumulative adjusted R^2
1 Positive face-to-face direct	-0.31	0.04	-7.22	<.001	0.134
2 Negative online vicarious	0.19	0.05	3.90	<.001	0.200
3 Positive online vicarious	-0.17	0.04	-3.94	<.001	0.217
4 Negative online direct	0.15	0.05	3.05	0.002	0.230

WHITE SAMPLE

	β	SE(β)	t	p	Cumulative adjusted R^2
1 Positive face-to-face direct	-0.21	0.05	-4.20	<.001	0.100
2 Negative face-to-face direct	0.21	0.05	3.91	<.001	0.202
3 Positive online vicarious	-0.13	0.05	-2.59	0.010	0.226
4 Negative face-to-face vicarious	0.17	0.05	3.38	0.001	0.240
5 Negative online vicarious	-0.13	0.05	-2.68	0.008	0.247
6 Negative online direct	0.16	0.06	2.85	0.005	0.255
7 Positive online direct	-0.12	0.06	-2.10	0.036	0.260

β = standardized coefficient
