# Supplementary material for: The contact hypothesis and the virtual revolution: Does face-to-face interaction remain central to improving intergroup relations?
Source: PLoS One. 2023 Dec 8;18(12):e0292831. doi: 10.1371/journal.pone.0292831 (PMC10707701; doi:10.1371/journal.pone.0292831)
Supplement: S9 File — (PDF) [file pone.0292831.s009.pdf]

SM6 Detailed mediated path results

Paths from contact to prejudice including individual and total path statistics

Path	β	SE	t	p
**Prejudice on Mediators**				
Prejudice on Anxiety	-0.158	0.059	-2.686	0.007
Prejudice on Realistic Threat	-0.171	0.059	-2.887	0.004
Prejudice on Symbolic Threat	0.110	0.063	1.756	0.079
**Mediators on Contact**				
Anxiety on Face-to-face positive	0.122	0.076	1.605	0.109
Anxiety on Face-to-face negative	0.038	0.066	0.577	0.564
Anxiety on Online positive	0.062	0.071	0.874	0.382
Anxiety on Online negative	-0.122	0.065	-1.866	0.062
Realistic Threat on Face-to-face positive	0.003	0.065	0.041	0.967
Realistic Threat on Face-to-face negative	0.008	0.063	0.128	0.898
Realistic Threat on Online positive	0.197	0.059	3.319	0.001
Realistic Threat on Online negative	-0.183	0.062	-2.970	0.003
Symbolic Threat on Face-to-face positive	-0.062	0.070	-0.886	0.376
Symbolic Threat on Face-to-face negative	0.074	0.064	1.148	0.251
Symbolic Threat on Online positive	-0.062	0.059	-1.051	0.293
Symbolic Threat on Online negative	0.113	0.061	1.855	0.064
**Mediated Paths Prejudice on Contact**				
Prejudice thru' Anxiety on Face-to-face positive	-0.021	0.015	-1.371	0.170
Prejudice thru' Anxiety on Face-to-face negative	-0.005	0.010	-0.547	0.585
Prejudice thru' Anxiety on Online positive	-0.010	0.013	-0.808	0.419
Prejudice thru' Anxiety on Online negative	0.017	0.012	1.559	0.119
Prejudice thru' Realistic Threat on Face-to-face positive	0.000	0.012	-0.039	0.969
Prejudice thru' Realistic Threat on Face-to-face negative	-0.002	0.012	-0.121	0.904
Prejudice thru' Realistic Threat on Online positive	-0.033	0.016	-2.129	0.033
Prejudice thru' Realistic Threat on Online negative	0.031	0.015	2.105	0.035
Prejudice thru' Symbolic Threat on Face-to-face positive	-0.006	0.009	-0.685	0.493
Prejudice thru' Symbolic Threat on Face-to-face negative	0.007	0.010	0.890	0.373
Prejudice thru' Symbolic Threat on Online positive	-0.007	0.010	-0.809	0.419
Prejudice thru' Symbolic Threat on Online negative	0.013	0.010	1.198	0.231
**Direct Paths Prejudice on Contact**				
Prejudice on Face-to-face positive	-0.120	0.064	-1.860	0.063
Prejudice on Face-to-face negative	-0.004	0.051	-0.077	0.939
Prejudice on Online positive	0.043	0.079	0.551	0.582
Prejudice on Online negative	0.057	0.049	1.163	0.245
**Total Paths Prejudice on Contact**				
Prejudice on Face-to-face positive	-0.147	0.072	-2.037	0.042
Prejudice on Face-to-face negative	-0.002	0.042	-0.057	0.954
Prejudice on Online positive	-0.006	0.074	-0.091	0.927
Prejudice on Online negative	0.122	0.055	2.202	0.028

β=standardized beta

Bootstrap estimates of confidence intervals for path statistics

Path	Lower .05%	Lower 2.5%	Lower 5%	Estimate	Upper 5%	Upper 2.5%	Upper .05%
**Prejudice on Mediators**							
Prejudice on Anxiety	-0.312	-0.276	-0.257	-0.158	-0.062	-0.042	-0.008
Prejudice on Realistic Threat	-0.338	-0.294	-0.272	-0.171	-0.079	-0.061	-0.024
Prejudice on Symbolic Threat	-0.065	-0.018	0.004	0.110	0.210	0.231	0.270
**Mediators on Contact**							
Anxiety on Face-to-face positive	-0.078	-0.027	-0.005	0.122	0.246	0.270	0.313
Anxiety on Face-to-face negative	-0.128	-0.092	-0.071	0.038	0.147	0.168	0.204
Anxiety on Online positive	-0.119	-0.074	-0.052	0.062	0.180	0.203	0.256
Anxiety on Online negative	-0.296	-0.250	-0.232	-0.122	-0.018	0.003	0.044
Realistic Threat on Face-to-face positive	-0.167	-0.128	-0.107	0.003	0.109	0.129	0.162
Realistic Threat on Face-to-face negative	-0.154	-0.118	-0.097	0.008	0.113	0.133	0.174
Realistic Threat on Online positive	0.048	0.081	0.100	0.197	0.296	0.315	0.352
Realistic Threat on Online negative	-0.339	-0.303	-0.283	-0.183	-0.079	-0.060	-0.025
Symbolic Threat on Face-to-face positive	-0.242	-0.200	-0.177	-0.062	0.051	0.074	0.120
Symbolic Threat on Face-to-face negative	-0.089	-0.049	-0.029	0.074	0.181	0.200	0.242
Symbolic Threat on Online positive	-0.224	-0.183	-0.165	-0.062	0.030	0.048	0.082
Symbolic Threat on Online negative	-0.040	-0.007	0.013	0.113	0.212	0.230	0.272
